# Two‐Dimensional Materials for Integrated Photonics: Recent Advances and Future Challenges

**DOI:** 10.1002/smsc.202000053

**Published:** 2021-02-19

**Authors:** Jianghong Wu, Hui Ma, Peng Yin, Yanqi Ge, Yupeng Zhang, Lan Li, Han Zhang, Hongtao Lin

**Affiliations:** ^1^ Key Lab. of Advanced Micro/Nano Electronic Devices & Smart Systems of Zhejiang College of Information Science & Electronic Engineering Zhejiang University Hangzhou 310027 China; ^2^ Key Laboratory of 3D Micro/Nano Fabrication and Characterization of Zhejiang Province School of Engineering Westlake University Hangzhou 310024 China; ^3^ Institute of Advanced Technology Westlake Institute for Advanced Study 18 Shilongshan Road Hangzhou 310024 China; ^4^ Institute of Microscale Optoelectronics Collaborative Innovation Centre for Optoelectronic Science & Technology International Collaborative Laboratory of 2D Materials for Optoelectronics Science and Technology of Ministry of Education Key Laboratory of Optoelectronic Devices and Systems of Ministry of Education and Guangdong Province College of Physics and Optoelectronic Engineering Shenzhen Key Laboratory of Micro-Nano Photonic Information Technology Guangdong Laboratory of Artificial Intelligence and Digital Economy (SZ) Shenzhen University Shenzhen 518060 P. R. China

**Keywords:** integrated photonics, light sources, optoelectronic devices, photodetectors, waveguide integrated modulators, 2D materials

## Abstract

With the development of novel optoelectronic materials and nanofabrication technologies, integrated photonics is a rapidly developing field that will promote the development and application of next‐generation photonic devices. In recent years, emerging two‐dimensional materials (2DMs) including graphene, transition metal dichalcogenides (TMDCs), black phosphorus (BP), and ternary compounds show many complementarities and unique characteristics over those of traditional optoelectronic materials including broadband absorption, ultrafast carrier mobility, strong nonlinear effects, and compatibility for monolithic integration. Herein, the recent progress on waveguide‐integrated active devices for a full photonic circuit based on 2DMs is reviewed. Both the development of nanofabrication techniques and the working mechanism of active photonic components based on 2DMs containing integrated light sources, waveguide‐integrated modulators, photodetectors, as well as some advanced 2DMs‐based optoelectronic devices are illustrated in detail. In the end, the existing challenges and perspectives on novel 2DMs‐integrated photonics are summarized and discussed.

## Graphical abstract

Two‐dimensional materials (2DMs) have recently emerged as an important class of photonic materials offering exceptional optical performance, which attracts increasing attention for a wide range of applications in integrated photonics. Herein, active optoelectronic devices based on 2DMs that could be the advanced components for future photonic circuits are reviewed.

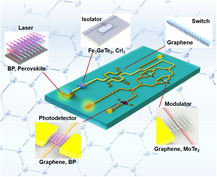

## 1 Introduction

Integrated photonics, which relies on semiconductor technology to shrink fiber or free‐space bulky optic circuits into on‐chip photonic devices, can significantly decrease the size, energy consumption, and costs of optical systems. It has been recognized as an advanced technology for communication, sensing, imaging, etc. Over the past decade, therefore, corresponding optoelectronic devices in integrated photonic systems based on Si, Ge, glass, LiNbO_3_, and III–V semiconductors (such as GaAs, InP, InGaAs) including lasers,^[^ ^1^, ^2^, ^3^, ^4^, ^5^ ^]^ couplers,^[^ ^6^, ^7^, ^8^ ^]^ Bragg gratings,^[^ ^9^, ^10^, ^11^ ^]^ waveguides,^[^ ^12^, ^13^, ^14^, ^15^, ^16^ ^]^ ring resonators,^[^ ^17^, ^18^, ^19^, ^20^ ^]^ modulators,^[^ ^21^, ^22^, ^23^, ^24^, ^25^ ^]^ and photodetectors^[^ ^26^, ^27^, ^28^, ^29^ ^]^ have been systematically explored and continuously optimized. Silicon and III–V photonics have been commercialized and are the backbone of current communication technology.^[^ ^30^, ^31^, ^32^, ^33^, ^34^ ^]^ However, with the rapid development of integration technology, the performance of each basic unit device gradually approached its fundamental limit. It is quite a challenge to propose novel device architectures and operational mechanisms to further boost the device components’ capabilities based on the current optoelectronic materials platform. For instance, 3 dB modulation bandwidth of the modulator based on bulk materials such as Si, III–V semiconductors, and LiNO_3_ is usually less than 200 GHz at the current telecommunication band.^[^ ^24^, ^35^, ^36^ ^]^ Free‐carrier dispersion is the primary mechanism of Si and III–V‐based optical modulators in which free‐carrier‐induced absorption is inevitable, resulting in degrading the optical modulation amplitude and even signal distortion in some applications. Since the discovery of graphene in 2004, 2DMs have attracted much attention in integrated photonics.^[^ ^37^, ^38^, ^39^ ^]^ For example, the ultrafast graphene‐loaded modulator has been obtained with a switching energy of 35 fJ and a switching time of 260 fs, which corresponded to 1.35 THz bandwidth.^[^ ^40^ ^]^ 2D WS_2_ has demonstrated a significantly higher carrier‐induced phase change relative to the absorption change than that in Si and III–V semiconductors, as substantially the insertion loss of the device can be reduced to a very low level.^[^ ^41^ ^]^ Thus, the seamless integration of 2DMs with ultracompact integrated photonic structures could probably pave the way for next‐generation on‐chip optoelectronic devices desiring higher device densities, lower power consumption, and faster response.^[^ ^40^, ^42^, ^43^ ^]^

**Figure 1:**
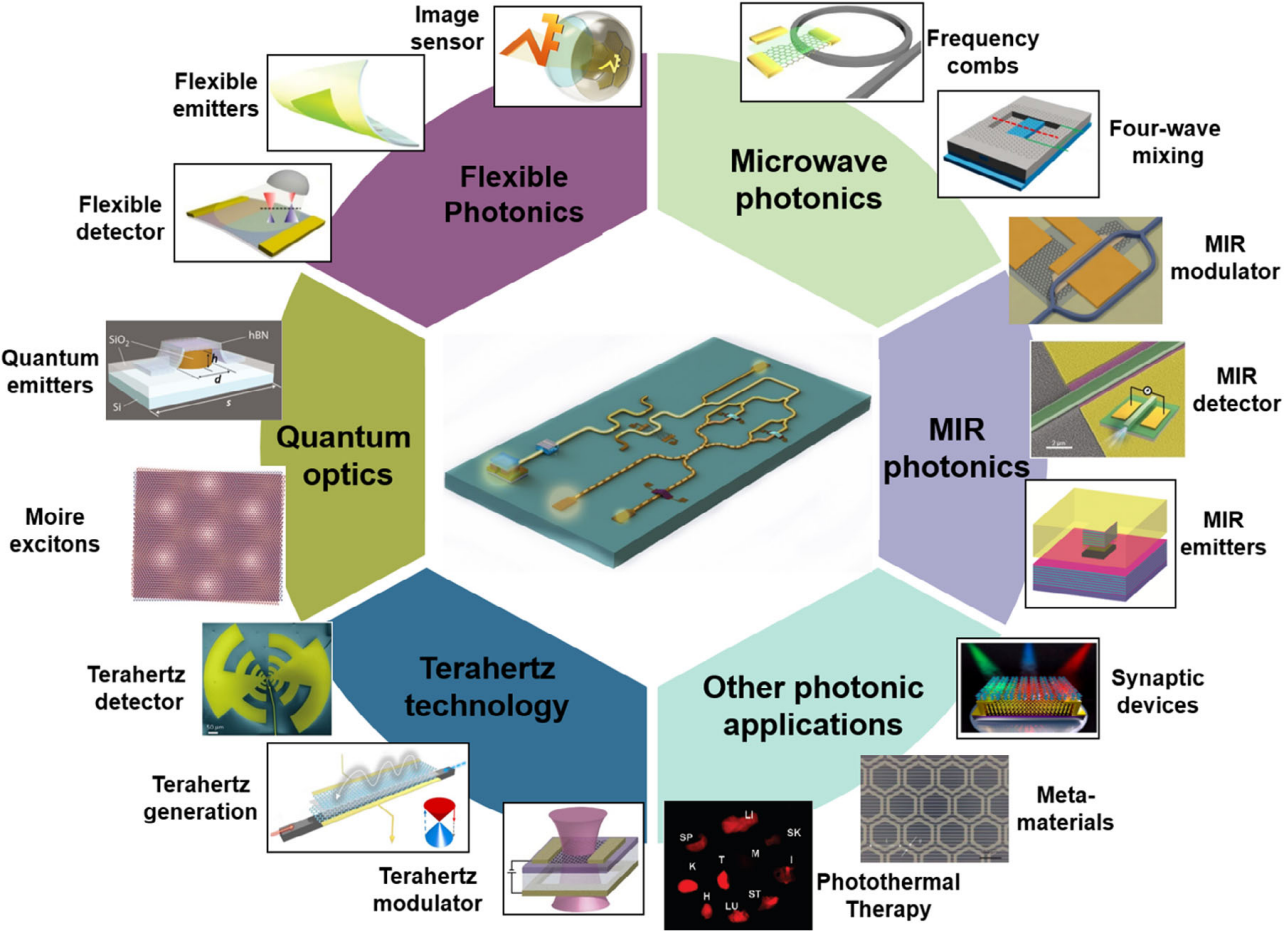
Some emerging photonic applications based on 2DMs have been demonstrated, including flexible photonics [flexible emitter (Reproduced with permission.^[^ ^75^ ^]^ Copyright 2012, Springer Nature), flexible detector (Reproduced with permission.^[^ ^74^ ^]^ Copyright 2014, American Chemical Society), and image sensor (Reproduced under the terms of the CC‐BY 4.0 license.^[^ ^76^ ^]^ Copyright 2017, The Authors, published by Springer Nature.)], MIR photonics [MIR emitter (Reproduced with permission.^[^ ^80^ ^]^ Copyright 2019, American Chemical Society), MIR modulator (Reproduced with permission.^[^ ^81^ ^]^ Copyright 2019, American Chemical Society), and MIR detector (Reproduced with permission.^[^ ^43^ ^]^ Copyright 2017, Springer Nature)], terahertz technology [terahertz generation (Reproduced with permission.^[^ ^78^ ^]^ Copyright 2017, Springer Nature), terahertz modulator (Reproduced with permission.^[^ ^52^ ^]^ Copyright 2012, Springer Nature), and terahertz detector (Reproduced with permission.^[^ ^103^ ^]^ Copyright 2012, Springer Nature)], microwave [frequency combs (Reproduced with permission.^[^ ^77^ ^]^ Copyright 2018, Springer Nature), and four‐wave mixing (Reproduced with permission.^[^ ^79^ ^]^ Copyright 2018, Chinese Laser Press)], quantum optics [quantum emitter (Reproduced with permission.^[^ ^73^ ^]^ Copyright 2018, Optical Society of America) and moire exciton (Reproduced with permission.^[^ ^72^ ^]^ Copyright 2019, Springer Nature)], and other photonic applications [synaptic devices (Reproduced with permission.^[^ ^82^ ^]^ Copyright 2020, American Chemical Society.), metamaterials (Reproduced with permission.^[^ ^83^ ^]^ Copyright 2012, Springer Nature), and photothermal therapy (Reproduced with permission.^[^ ^84^ ^]^ Copyright 2010, American Chemical Society)].

In addition, emerging integrated photonic applications such as flexible photonics,^[^ ^44^, ^45^, ^46^, ^47^ ^]^ mid‐infrared (MIR) photonics,^[^ ^48^, ^49^, ^50^ ^]^ terahertz technology,^[^ ^51^, ^52^, ^53^ ^]^ quantum optics,^[^ ^54^, ^55^, ^56^ ^]^ microwave photonics,^[^ ^57^, ^58^, ^59^ ^]^ etc. request more advanced material properties such as the novel and unique optoelectronic characteristics, large mechanical flexibility, and wide compatibility with nanofabrication processing technologies. 2DMs containing insulator (h‐BN),^[^ ^60^, ^61^ ^]^ semiconductors with various bandgaps (TMDCs, black phosphorous [BP]),^[^ ^62^, ^63^, ^64^ ^]^ semimetal (graphene, WTe_2_),^[^ ^65^, ^66^, ^67^, ^68^ ^]^ and metal (CrI_3_)^[^ ^69^, ^70^ ^]^ could provide a wide range of options for optoelectronic devices and fulfill the request of emerging photonic applications (**Figure** **1**)^[^ ^43^, ^71^, ^72^, ^73^, ^74^, ^75^, ^76^, ^77^, ^78^, ^79^, ^80^, ^81^, ^82^, ^83^, ^84^, ^85^ ^]^ because of their unique optical, electronic, thermal, and mechanical features.^[^ ^86^, ^87^, ^88^, ^89^, ^90^ ^]^ First, 2DMs usually show better mechanical flexibility than their bulk counterparts due to the atomic‐level thickness; thereby, 2DMs are good choices for flexible photonics. For instance, flexible photodetectors based on different 2DMs have already been studied and exhibited good performance.^[^ ^91^, ^92^, ^93^, ^94^ ^]^ Second, these 2DMs with zero and narrow bandgap, such as graphene, BP, and PtSe_2_, are good candidates for MIR photonics,^[^ ^95^, ^96^, ^97^, ^98^ ^]^ which plays an important role in thermal imaging, infrared homing, and spectroscopic sensing.^[^ ^99^ ^]^ Up to now, several 2DMs MIR devices such as mode‐locked pulses,^[^ ^100^ ^]^ modulators,^[^ ^81^ ^]^ and photodetectors^[^ ^101^ ^]^ have been designed. Third, 2DMs such as graphene with a gapless bandgap are good choices to promote terahertz technology that is significant for noninvasive and nondestructive detection. Some representative devices have been fabricated to demonstrate the generation,^[^ ^78^, ^102^ ^]^ modulation,^[^ ^52^ ^]^ and detection^[^ ^103^, ^104^ ^]^ of terahertz signal based on graphene. Fourth, the strong optical response of 2DMs is another advantage.^[^ ^105^, ^106^, ^107^, ^108^ ^]^ Till now, nonlinear phenomena of 2DMs including ultrafast nonlinear absorption,^[^ ^109^, ^110^, ^111^ ^]^ thermo–optic (TO) nonlinearities,^[^ ^112^ ^]^ second‐harmonic generation (SHG),^[^ ^113^, ^114^, ^115^ ^]^ and third‐order nonlinear optical response^[^ ^116^, ^117^ ^]^ have been observed, which are important for next‐generation nonlinear photonic circuits. Fifth, quantum emitters based on 2DMs,^[^ ^118^ ^]^ moire excitons in van der Waals’ heterojunctions of 2DMs,^[^ ^119^, ^120^ ^]^ and gate‐tunable frequency combs based on graphene/Si_3_N_4_ microresonator^[^ ^77^ ^]^ have been realized, which bring about a new possibility for quantum optics. Sixth, despite being atomically thin, many 2D materials show large light absorption over a broad wavelength range,^[^ ^88^, ^121^ ^]^ which is partially due to the strong quantum confinement in the direction perpendicular to the 2D plane. Seventh, 2DMs, exhibiting high carrier mobility,^[^ ^122^ ^]^ can be applied to photodetectors and modulators with fast response speed.^[^ ^37^, ^123^, ^124^ ^]^ Taking graphene as an example, it possesses ultrafast carrier mobility (2 × 10^4^ cm^2^ V s^−1^ at room temperature).^[^ ^125^ ^]^ Last but not least, van der Waals‐stacked heterojunctions and homojunctions of 2DMs obtained by direct transfer can bring about novel physical phenomena for the devices’ excellent performance.^[^ ^126^, ^127^, ^128^, ^129^, ^130^ ^]^ Taking graphene as an example, bilayer graphene with magic‐angle superlattices can work as both a superconductor and an insulator at a different angle.^[^ ^131^, ^132^ ^]^

In the past few years, integrated light sources,^[^ ^133^ ^]^ waveguide‐integrated modulators^[^ ^37^, ^43^, ^134^ ^]^ and photodetectors^[^ ^42^, ^135^ ^]^ based on 2DMs, have been realized, benefiting from the properties mentioned earlier. Based on the current research status and potential applications of 2DMs, therefore, the rapid development of integrated photonic technology promotes the demand for exceptional optoelectronic 2D materials. The introduction of them would lead to the unconventional tuning and detuning mechanism in integrated photonic devices, which could not only be the key to break the theoretical limits of current device architectures but also facilitate some creative applications of integrated photonics.

Although 2DMs have emerged as a rising star in the field of integrated photonics, there are still some challenges to resolve for the practical use of 2DMs‐based devices. For example, the electrically pumped laser is essential in practical applications, but currently reported lasers based on TMDCs are all optically pumped, whereas most of them emit the laser beam perpendicular to the surface and cannot be easily coupled into the planar photonic circuits. Moreover, only a limited number of studies focus on novel waveguide‐integrated optoelectronic devices such as isolators, modulators, and single‐photon detectors based on 2DMs exhibiting excellent performance.

In this Review, the recent progress in on‐chip integrated optoelectronic devices based on 2DMs has been summarized. First of all, different photonic integration technologies with 2DMs for rapid prototyping and wafer‐scale manufacturing are discussed and compared. The latest advances in the development of design principles and functionalities for novel 2DMs‐based active optoelectronic devices, including integrated light sources, waveguide‐integrated modulators, and photodetectors, are reviewed in the next three sections. Also, some advanced integrated optoelectronic devices based on quantum, phase‐change, and magneto‐optical 2DMs are introduced in the following section. Finally, the last section concludes the remaining challenges and provides perspectives on the further development of novel 2DMs‐based integrated devices.

## 2 Fabrication Strategy

The fabrication process has a severe impact on the performance of optoelectronic devices based on 2DMs because those ultrathin 2DMs are fragile. To fabricate devices based on 2DMs with less damage, one solution is transferring 2DMs onto a prepatterned substrate. Different types of waveguide‐integrated photodetectors and modulators have been designed and optimized through this method. In 2011, Si waveguide‐integrated graphene modulators were reported.^[^ ^37^ ^]^ Although transferring 2DMs onto a prepatterned waveguide is a commonly used method, there do exist some limitations. On the one hand, 2DMs are ultrathin, thereby the out‐of‐plane stress induced by uneven prepatterned optical waveguides can disrupt the integration of 2DMs, resulting in degradation in device performance.^[^ ^136^ ^]^ To prevent 2DMs from rupturing at the waveguide edge on an uneven substrate, researchers have to take additional fabrication procedures. For example, backfilling of the prepatterned substrate with SiO_2_ has been proven to be necessary for high‐performance photodetectors.^[^ ^38^ ^]^ On the other hand, the overlap between the optical field in a waveguide with 2DMs is limited if 2DMs are on top of the waveguide structure.^[^ ^137^ ^]^ Moreover, it's challenging to transfer complicated structures such as heterojunction and prepatterned structures onto the substrates with waveguides, limiting the capability to achieve more functional devices.

Limited by the problems as described earlier, depositing optical dielectric films onto 2DMs seems to be a good solution, and some research groups have tried this approach. However, this technique presents some problems as well. On the one hand, it is difficult to initiate the nucleation and deposition of dielectric films with their optical thickness on the chemically inert 2DM surface by atomic layer deposition (ALD),^[^ ^138^ ^]^ a pretty mild technique for thin‐film deposition. On the other hand, direct damage to 2DMs is inevitable if adopting other thin‐film deposition techniques including plasma‐enhanced chemical vapor deposition (PECVD), magnetron sputtering, and electron beam evaporation, in which 2DMs might be bombarded by the generated plasma and high‐energy atoms and ions. For example, the defectpeak at 1350 cm^−1^ appears in Raman spectrum after the deposition of TiO_2_ and SiO_2_ onto graphene by electron beam evaporation, leading to a decrease in carrier mobility in grapheme.^[^ ^43^ ^]^

To develop a less‐destructive fabrication technology, some research groups have been trying to explore new approaches. One such method is to pattern spin‐coated polymers directly upon 2DMs, but the device performance and functionality might be limited by polymers’ low refractive index.^[^ ^139^ ^]^ Recently, chalcogenide glass (ChG), an optical dielectric material consisting of S, Se, and/or Te, provides several significant advantages for nondestructive photonic integration with 2DMs. First, ChG possesses unique optical characteristics including broadband transparency, a tunable high refractive index ranging from 2 to 3.5 and large Kerr nonlinearity^[^ ^140^, ^141^ ^]^ and can be a good material candidate for integrated photonics. Second, the thermal deposition temperature of ChG is usually below 600 K in vacuum with the substrate temperature at room temperature,^[^ ^142^ ^]^ causing minimal damage to 2DMs. It has been proved by Raman spectra that ChG has had little impact on MoS_2_, InSe, BP, and h‐BN.^[^ ^43^ ^]^ Benefitting from this fabrication technology, 2DMs can be placed at the location where device performance will be maximized, more complicated devices can be designed, such as waveguide‐integrated photodetectors and modulators based on heterojunctions. Apparently, ChG photonic structures can be monolithically integrated with 2DMs on flat substrates, without having the chance to introduce cracks in 2DMs at the waveguide edge. In addition, for 2DMs with in‐plane anisotropy, it would be easier to achieve high‐performance devices by monolithically defining the photonic structures and thereby, aligning well with the desired crystal orientation in 2D materials. However, a shortcoming is that this fabrication is not compatible with current complementary metal–oxide semiconductor (CMOS) technology. Notably, Ge_2_Sb_2_Te_5_, a kind of ChG, is a basic material for phase‐change switch,^[^ ^143^ ^]^ which may provide the possibility for ChG being compatible with COMS backend integration technology in the future.

## 3 Light Sources

Integrated light sources are vital components of integrated photonics. The light emission is mainly induced by the recombination of free electron–hole pairs in semiconductors. Electrons in semiconductors can transit from the ground state to the high‐level energy band through effective optical, electrical, or thermal excitation. Those excited free electrons in the high‐level energy band in semiconductors, with ultrashort lifetime, can recombine with those unstable holes in the ground state, leading to emitting photons. Generally, integrated light sources including light‐emitting diodes (LEDs) and lasers can be categorized by different radiation types. An LED is usually excited electrically based on spontaneous emission, whereas a laser is induced by stimulated emission which is typically more difficult to achieve.^[^ ^144^, ^145^, ^146^, ^147^ ^]^ First, the population inversion in gain materials is essential under excitation. For example, the condition for population inversion in a three‐level system can be expressed as

(1) $$W_{13}>A_{21}$$

where $W_{13}$ is the probability of stimulated transition and $A_{21}$ is the probability of spontaneous emission, which mainly depends on gain materials. Second, a resonant cavity is necessary to unify the phase and direction of the emitted photons, and the number of generated photons in a cavity must be larger than that of the photon loss due to the cavity decay. Light power ($P_{\text{round}}$) that propagates in the cavity after a round trip can be given as

(2) $$P_{\text{round}}=P_{0}\cdot \text{exp}[2\cdot (G\cdot L_{c}-\delta )]$$

where $P_{0}$ is the initial power, *G* is the gain factor, $L_{c}$ is the length of the cavity, and *δ* is the total loss. One condition for lasing is $G\geq \delta /L_{c}$, which is mostly influenced by the optical resonator cavity. In the following paragraphs, light sources based on 2DMs are presented in detail. Several parameters including the lasing threshold, full width at half maximum (FWHM), quality factor (*Q*) (the quotient between the stored energy and the lost energy in unit time in the cavity), and beta factor (*β*) (the percentage of spontaneous emission coupled to the resonant mode) that characterize the device performance are discussed and compared.

**Figure 2:**
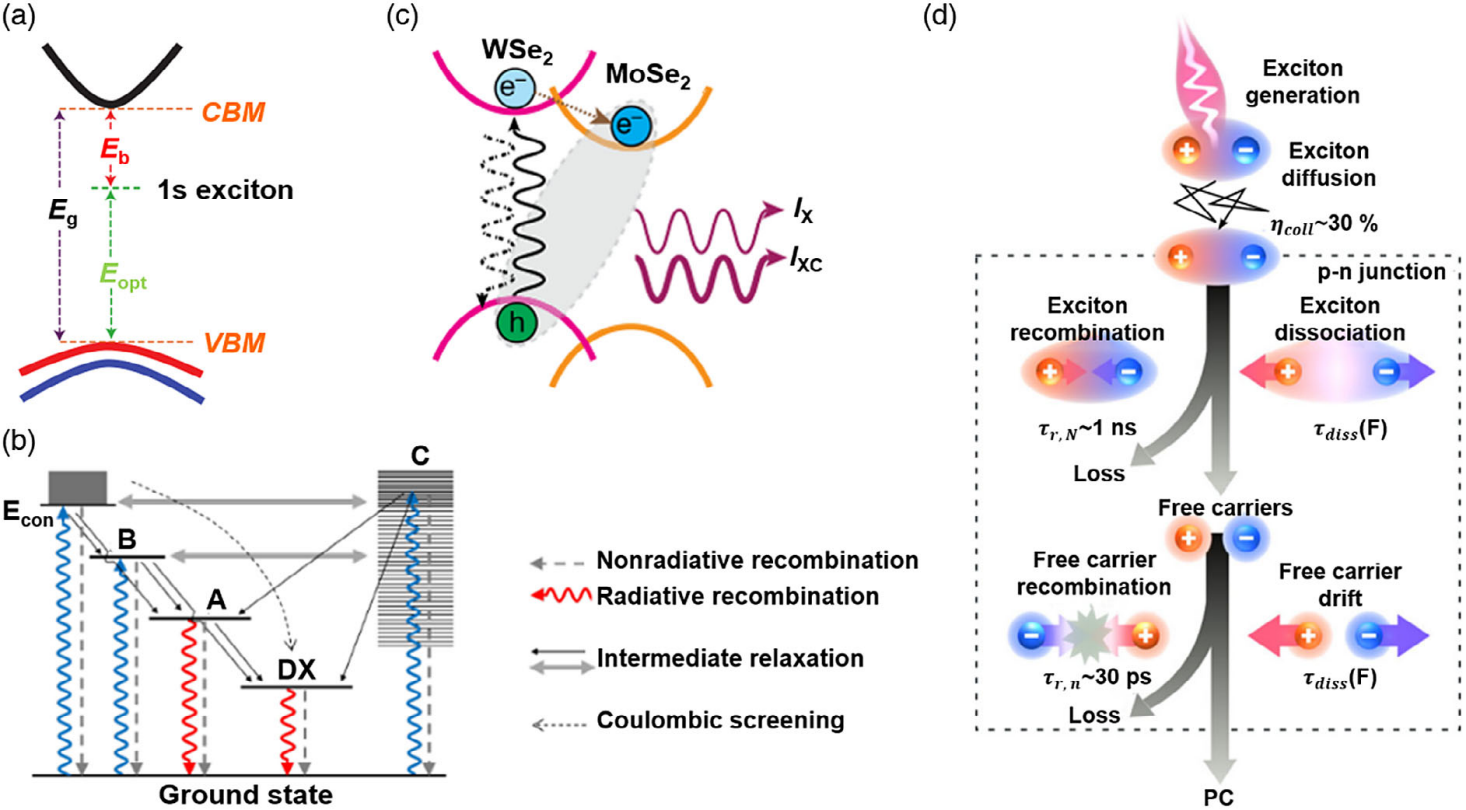
a) The band structure of monolayer MoSe_2_. Reproduced with permission.^[^ ^148^ ^]^ Copyright 2014, Springer Nature. b) The schematic level picture demonstrating the generation–relaxation–recombination of photoinduced carriers, where A, B, C are excitons with a different energy, DX is the defect‐bound exciton. Reproduced with permission.^[^ ^150^ ^]^ Copyright 2017, American Physical Society. c) The illustration of the energy band of WSe_2_/MoSe_2_ heterojunction. Reproduced with permission.^[^ ^151^ ^]^ Copyright 2019, Springer Nature. d) The concrete process of excitons recombination and dissociation in the WSe_2_ PN junction under the in‐plane electrical field. Reproduced under the terms of the CC‐BY 4.0 license.^[^ ^153^ ^]^ Copyright 2018, The Authors, published by Springer Nature.

Light sources based on 2DMs have drawn much attention because of their intriguing features, such as a large exciton binding energy. **Figure** **2**a shows the energy band diagram of monolayer MoSe_2_, where *E*_g_, *E*_opt_, and *E*_b_ are electronic bandgap (the difference between the minimum conduction band and maximal valence band), optical bandgap (the least bandgap for exciton emission), and exciton binding energy ($E_{b}^{X}=E_{g}-E_{\text{opt}}$), respectively.^[^ ^148^ ^]^ The exciton binding energy is greater than 0.5 eV for most monolayer TMDCs,^[^ ^149^ ^]^ which partially results from strong Coulomb screening and weak screening effect. Therefore, stable exciton‐enhanced emissions can be obtained at room temperature, and excitonic emission of 2D TMDCs plays an important role in their emission spectra. More concrete processes of transition and relaxation pathways of photoinduced carriers are shown in Figure 2b.^[^ ^150^ ^]^ Usually, electrons that are bound at the ground state can be excited to the high‐level energy band by several pathways in TMDCs and go back to the ground state by recombination with holes. Before recombination, intermediate relaxation and coulombic screening usually occur, leading to the lower energy of emission spectra. Among these relaxation pathways, exciton emission (A) contributes to the primary photoluminescence (PL) signal of monolayer TMDCs. Besides intralayer excitons of monolayer TMDCs, interlayer exciton in a heterojunction is an intriguing phenomenon. A type‐II energy band consisting of WSe_2_ and MoSe_2_ is shown in Figure 2c.^[^ ^151^ ^]^ In such a structure, photoinduced electrons’ relaxation from the high‐level conduction band to the low‐level conduction band occurs because of the energy difference between the conduction bands of two different 2DMs. For example, the energy of the bottom conduction band of WSe_2_ (*E*_C,WSe2_) is higher than that of MoSe_2_ (*E*_C,MoSe2_); thereby, photoinduced electrons’ relaxation from *E*_C,WSe2_ to *E*_C,MoSe2_ occurs. Moreover, radiative recombination of electrons at *E*_C,MoSe2_ and holes at the top valence band of WSe_2_ (*E*_V,WSe2_) occurs, leading to emitting photons with a lower energy than that of intralayer excitons of both WSe_2_ and MoSe_2_. In addition, the PN homojunction of 2DMs is another structure for light sources. As for light sources based on 2DMs, exciton–exciton annihilation that is about two orders of magnitude higher than that in bulk semiconductors is a significant factor to assess quantum efficiency.^[^ ^152^ ^]^ As shown in Figure 2d, excitons recombination and dissociation relating to exciton–exciton annihilation of monolayer WSe_2_ PN junction were studied, indicating that excitons dissociation could be explained by 2D Wannier–Mott exciton model.^[^ ^153^ ^]^ This result is important for both the design of light sources and photodetectors.

**Figure 3:**
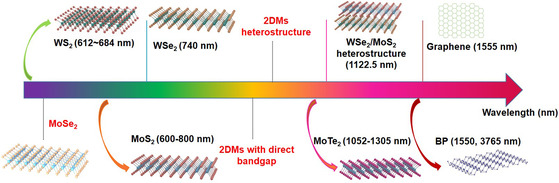
2D gain materials and their corresponding emitting wavelength reported in the literature. Dotted lines and red words represent potential gain materials.

**Figure 4:**
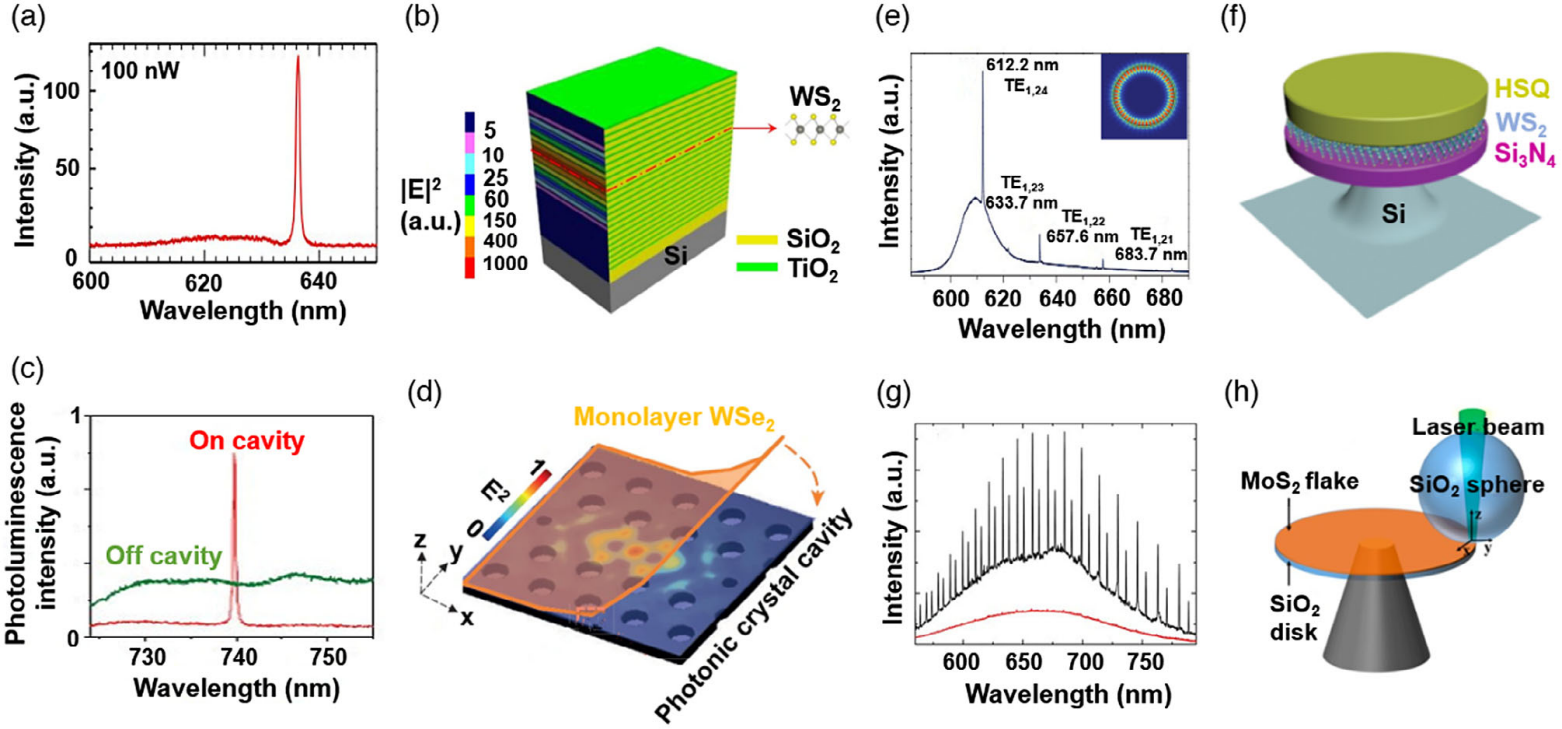
a) The emission spectrum of a WS_2_‐based VCSEL and b) the corresponding device schematic and electric field distribution. a,b) Reproduced under the terms of the CC‐BY 4.0 license.^[^ ^176^ ^]^ Copyright 2017, The Authors, published by Springer Nature. c) The emission spectrum of WSe_2_ on a photonic crystal cavity and d) the corresponding device structure and electric field distribution. c,d) Reproduced with permission.^[^ ^155^ ^]^ Copyright 2015, Springer Nature. e) The emission spectrum of WS_2_‐based whispering gallery modes (WGMs) cavity laser, the inset shows the electric field distribution in the cavity, and f) the corresponding device diagram (Si_3_N_4_/WS_2_/HSQ structure). e,f) Reproduced with permission.^[^ ^157^ ^]^ Copyright 2015, Springer Nature. g) The emission spectrum of MoS_2_‐based WGM cavity laser, the inset shows the electric field distribution in the cavity, and h) the corresponding device schematic (SiO_2_ nanodisk/MoS_2_/SiO_2_ nanosphere sandwich structure). g,h) Reproduced with permission.^[^ ^158^ ^]^ Copyright 2015, American Chemical Society.

Optical materials are critical for light sources, and a part of 2D gain materials used for light sources is shown in **Figure** **3**. Among the discovered 2DMs, monolayer TMDCs have shown great potential in the application of lasers and LEDs.^[^ ^154^, ^155^ ^]^ These TMDCs’ bandgaps transform from a direct (monolayer) to indirect one (multilayer), and most monolayer TMDCs have a direct bandgap ranging from 1 and 2 eV. For example, the bandgaps of monolayer MoS_2_, MoSe_2_, WS_2_, and WSe_2_ are 1.89, 1.64, 1.96, and 1.6 eV, respectively.^[^ ^156^ ^]^ In 2015, three different light sources based on monolayer WSe_2_, WS_2_, and four‐layer MoS_2_ were demonstrated, and the emitting wavelength (shown in **Figure** **4**) ranges from 600 to 800 nm.^[^ ^155^, ^157^, ^158^ ^]^ Notably, four‐layer MoS_2_ after oxygen‐plasma treatment exhibiting direct bandgap^[^ ^159^ ^]^ possesses a weaker effect in Auger recombination, leading to a higher quantum efficiency at a high carrier concentration. Recently, microscopic gain calculations of MoS_2_, WS_2_, and WSe_2_ theoretically verified the availability of 2DMs‐based light sources.^[^ ^160^ ^]^ The emission wavelength of light sources based on MoTe_2_ can be up to 1132^[^ ^161^ ^]^ and 1305 nm^[^ ^162^ ^]^ because monolayer MoTe_2_ has a relatively small direct bandgap of ≈1.1 eV. About 1305 nm so far is the longest lasing wavelength based on monolayer TMDCs, which is in the center of optical communications “O band.” In addition to TMDCs, graphene, demonstrating a high saturation current density, good stability at a high temperature, and ultrafast heating and cooling speed, is an ideal material for thermal emitters. A recent study showed that narrow near‐infrared (NIR) emission (telecommunication band) could be observed in graphene/photonic‐crystal nanocavity structure, and the emission wavelength is tunable by an externally applied voltage.^[^ ^163^ ^]^ Under 2000 K, those heated carriers could first reach equilibrium with optical phonons and dissipate to the planar silicon photonic‐crystal nanocavity substrate (at a lower temperature) because of the weaker interaction of acoustic phonon scattering in graphene.^[^ ^164^, ^165^ ^]^ In addition, thin‐film BP, possessing the direct and moderate bandgap (≈0.33 eV), can be used for NIR and even MIR light sources.^[^ ^80^, ^166^ ^]^ Although BP is unstable in the ambient condition, surface passivation or chemical modification can effectively prevent BP from degradation.^[^ ^167^ ^]^ Recently, a surface‐emitting MIR laser (≈3765 nm) based on BP at room temperature was reported.^[^ ^80^ ^]^

Comparing with monolayer 2DMs, van der Waals‐stacked heterojunctions constructed by different types of 2DMs have drawn much attention in recent years because of their advantages as alternative light‐emitting materials.^[^ ^72^, ^168^, ^169^, ^170^, ^171^ ^]^ First, the energy of interlayer excitons in heterojunctions (type‐II band alignment) can be smaller than that of intralayer excitons, leading to a redshift in emission. Recent work showed that the emission wavelength of MoS_2_/WSe_2_ heterojunction was up to 1122.5 nm^[^ ^172^ ^]^ that is longer than that of the intralayer excitons’ emission wavelength. Second, interlayer excitons (type‐II band alignment) possess a longer lifetime than intralayer excitons that promote population inversion. A recent interesting study showed spatially coherent NIR lasing emission based on WSe_2_/MoSe_2_ heterojunction,^[^ ^151^ ^]^ in which moiré lattices in the heterojunction^[^ ^173^ ^]^ probably enhanced phase space density and decreased nonradiative loss due to localization of interlayer excitons. Third, a high quantum efficiency can be obtained in type‐III heterojunctions.^[^ ^174^ ^]^ Last but not the least, studying light sources based on van der Waals heterojunctions could bring about new opportunities, because PN junction is an essential structure for the electrically pumped laser.

A low‐loss optical resonator cavity with a high Purcell factor is another critical factor for lasing. Up to now, several structures, including 1D and 2D photonic‐crystal nanocavities, microdisks and microsphere cavities, and distributed Bragg reflectors (DBRs) cavity, have been applied for lasers based on 2DMs. Among these cavities, DBRs are the essential structures for vertical‐cavity surface‐emitting lasers (VCSELs)^[^ ^175^ ^]^ that possess several advantages such as low energy consumption, high‐yield fabrication, and fast modulation capability. VCSELs based on 2DMs were first reported in 2017. This WS_2_‐based VCSEL through optical pumping was reported (Figure 4b), and the vertical cavity with a quality factor (*Q*) of 1800 was achieved, but *Q* was reduced to 640 after introducing WS_2_.^[^ ^176^ ^]^ This dramatic reduction of *Q*, usually observed in other 2DMs‐based laser, is mainly due to the absorption from 2DMs.^[^ ^155^ ^]^ Usually, a significant difference of refractive indices between 2DMs and an upper dielectric layer is necessary to obtain strong optical confinement, and surface passivation is also critical before depositing the dielectric layer. In commercial applications, VCSEL technology is not feasible for MIR emission, but recent literature reported a BP‐based MIR VCSEL under optical pumping.^[^ ^80^ ^]^ Comparing with traditional VCSELs, 2DMs can be transferred onto various dielectric mirrors without worrying too much about the lattice mismatch.

Photonic‐crystal cavity is another optical resonator cavity that can capture light in the defect of the photonic crystal when the periodicity is broken, playing a significant role in enhancing light–matter interaction.^[^ ^177^, ^178^ ^]^ The thickness of the cavity has a severe influence on *Q*, leading to the different emission characteristics of semiconductors. For example, TMDCs on a photonic‐crystal cavity with a thickness of 180 nm can emit enhanced PL, but the lasing emission can be induced when the thickness decreases from 180 to 125 nm because of the better thickness‐to‐lattice‐constant ratio and enhanced sidewall verticality.^[^ ^155^, ^179^ ^]^ Up to now, the photonic crystal cavity is the most commonly used structure for 2DMs‐based lasers,^[^ ^155^, ^161^, ^172^, ^180^ ^]^ and several different photonic‐crystal surface‐emitting lasers based on 2DMs have already been achieved through optimizing the device structures.

WGM resonators have also drawn much attention in high‐performance lasers in the past few decades.^[^ ^181^, ^182^ ^]^ WGM resonators can confine the incident light in the circular boundary of the cavities by continuous internal reflection. Up to now, several microstructures with different morphologies such as microdisks, microrings, microtoroids, microbottles, and hemispheres have been demonstrated as resonant cavities for lasing emission.^[^ ^183^ ^]^ In recent years, several lasers based on 2DMs, integrating with WGM microcavities, have been realized. WS_2_ was embedded between the Si_3_N_4_ microdisk and hydrogen silsesquioxane (HSQ), achieving the lasing emission at a low temperature (Figure 4f).^[^ ^157^ ^]^ As shown in Figure 4e, this WGM cavity with a *Q* of ≈2604 not only possessed strong optical confinement within the two microdisks but also prevented 2DMs from reaction and contamination. Mode competition was avoided by the separation of the resonance mode in a small volume, resulting in a low threshold. By inserting four‐layer MoS_2_ into two different WGMs (SiO_2_ microsphere and microdisks), lasing emission has been observed at room temperature, which is the first room‐temperature laser based on 2DMs (Figure 4h). Optical intensity is trapped within the boundary of the microsphere and microdisk; thereby, the most vigorous optical emission can be observed in the contact area between two different WGMs. Because of the improved electrical field distribution in this asymmetric cavity (the highest *Q* of 3300), the derived mode volume decreases.^[^ ^158^ ^]^

**Figure 5:**
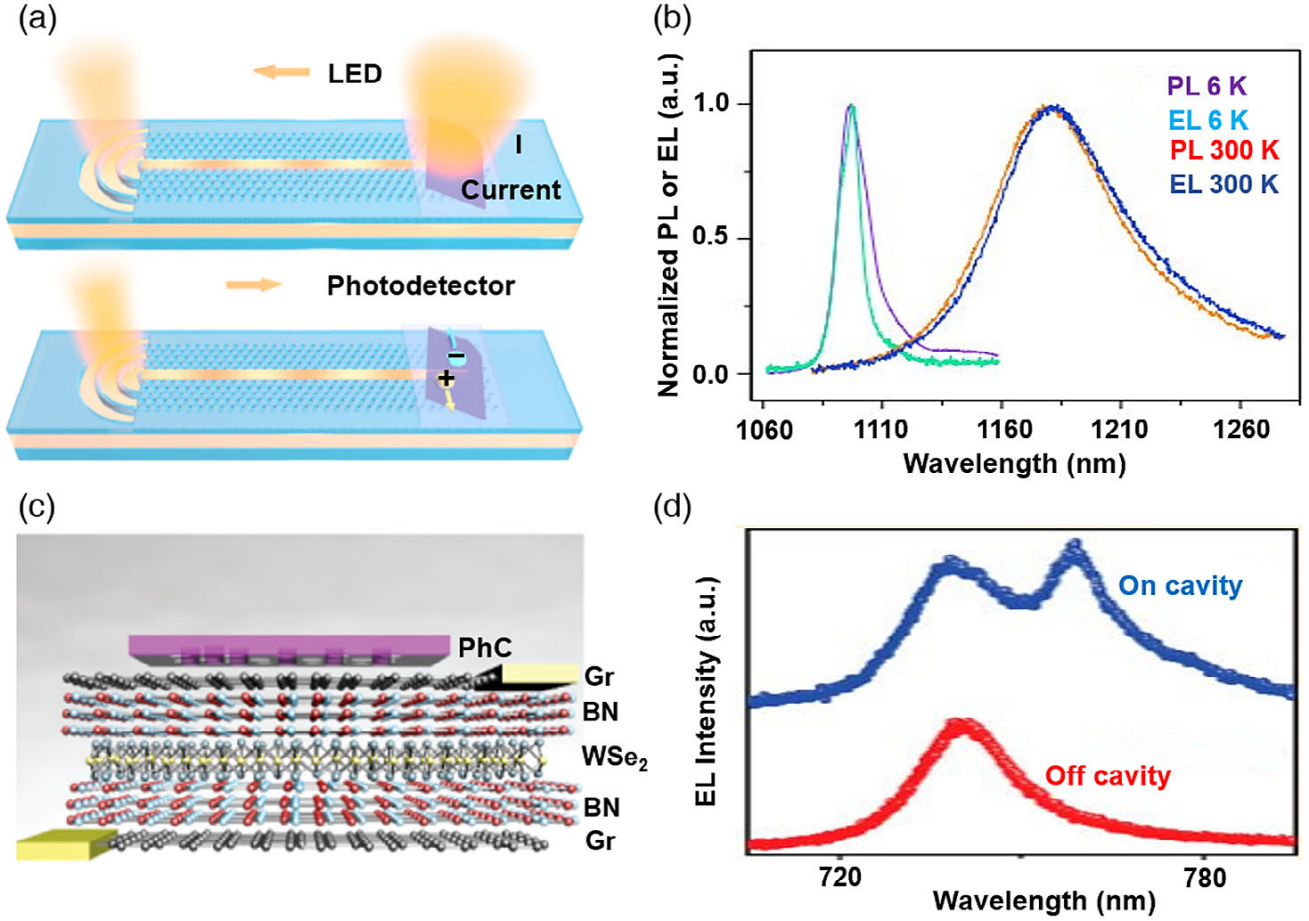
a) The Si waveguide‐integrated MoTe_2_‐based LED and photodetector. b) Electroluminescence (EL) and PL spectrum of the Si waveguide‐coupled device under different temperatures. a,b) Reproduced with permission.^[^ ^133^ ^]^ Copyright 2017, Springer Nature. c) Device architecture of cavity‐coupled LED. d) EL spectrum showing on and off nanocavity. c,d) Reproduced with permission.^[^ ^188^ ^]^ Copyright 2016, American Chemical Society.

Moreover, the ultimate goal for integrated light sources is that it can be electrically pumped. Up to now, electrically driven LEDs based on 2DMs have been demonstrated,^[^ ^184^, ^185^ ^]^ but electrically pumped lasers based on 2DMs have not been achieved yet. As for in‐plane LEDs, the lateral monolayer WS_2_ PN junction emitting electroluminescence (EL) at the red spectral region has been demonstrated.^[^ ^186^ ^]^ The vertical PN junction consisting of n‐type few‐layer MoS_2_ and p‐type monolayer WSe_2_ worked as an electrically pumped broadband light emitter with an external quantum efficiency (EQE) of 12%.^[^ ^187^ ^]^ To realize a complete optical link, both the light source and detector should be integrated on the same platform. Recently, Bie et al. from MIT showed the waveguide‐integrated NIR light emission and detection based on a PN junction of bilayer MoTe_2_ (**Figure** **5**a,b).^[^ ^133^ ^]^ Another literature reported van der Waals heterojunctions consisting of graphene, h‐BN, and WSe_2_, and coupling with a photonic crystal cavity on top of the device exhibited some intriguing light emission phenomena (Figure 5c).^[^ ^188^ ^]^ For instance, EL intensity was increased by four‐fold with a broadband emission wavelength region and a modulation speed of ≈1 MHz after introducing the photonic crystal cavity. Single‐mode and highly linear polarized (84%) EL was achieved as well (Figure 5d).

Light sources based on 2DMs have made significant progress in the past decade, which is shown in **Table** **1**. Although on‐chip integrated light sources based on 2DMs showed several advantages, there are many critical challenges for practical implementation in integrated photonics. As for LEDs, most LEDs based on 2DMs usually possess an EQE that is below 10%, which is much lower than those LEDs based on traditional III–V materials. Another challenge is that the emission wavelengths of LEDs were limited to the range from orange to NIR, and LEDs emitting other wavelengths are still waiting for further exploration. As for lasers, first, the electrically pumped laser based on 2DMs has not been demonstrated. Second, to obtain a low lasing threshold, photonic cavities with a high Purcell factor are necessary but difficult to fabricate without causing damage to 2DMs. Third, population inversion, an essential condition for lasers, is a challenge for TMDCs. For example, because of the high densities of states attributed to the large effective mass of carriers in MoS_2_,^[^ ^189^, ^190^ ^]^ a high carrier concentration up to 5 × 10^18 ^cm^−3^ was required to tune the quasi‐*E*_F_ so that population inversion can be realized.^[^ ^158^ ^]^ Last but not the least, the inhomogeneous broadening of 2DMs partially induced by surface defect states could also reduce the gain and influence the device performance.

**Table 1:** Figures of merit in typical 2DMs‐based light sources. (NR: not reported, CW: continuous wave)

Material and device structure	Lasing wavelength [nm]	Pump source	Pump power [W cm^−2^]	FWHM [nm]	Beta factor	Operational temperature	Ref
WSe_2_ on a photonic‐crystal cavity	≈740	CW	≈1	0.30	0.19	80–160 K	[155]
WS_2_ in a microdisk resonator	≈612, ≈634, ≈658, ≈684	Pulse	0.02	0.24~0.28	0.5	10 K	[157]
MoS_2_ between the microdisk and microsphere cavity	600–800 (multiple lasing peak)	CW	>1000	<0.30	NR	Room temperature	[158]
WS_2_ in a half‐wavelength‐thick cavity	≈637	CW	0.44	≈0.7	NR	Room temperature	[176]
MoTe_2_ on a Si photonic‐crystal cavity	≈1052, ≈1132	CW	6.6	≈0.20	0.1	Room temperature	[161]
MoTe_2_ on a Si photonic‐crystal cavity	≈1250, 1305	CW	1500	≈0.49	0.5	Room temperature	[162]
WSe_2_/MoS_2_ heterojunction on a photonic‐crystal cavity	≈1122.5	CW	≈54 uW	2.65–2.85	0.27	Room temperature	[172]

## 4 Waveguide‐Integrated Modulators

**Figure 6:**
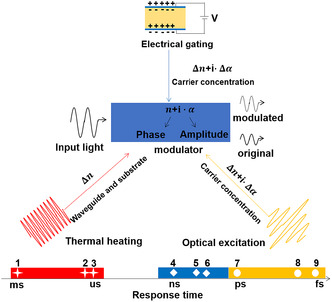
The tunable refractive indices of 2DMs induced by the excitation of electric, optical, and thermal fields. The response time of 2DM modulators modulated by thermal, electrical, and optical signals is plotted in red, orange, and green, respectively, based on representative experimental results (1,^[^ ^348^ ^]^ 2,^[^ ^219^ ^]^ 3,^[^ ^349^ ^]^ 4,^[^ ^37^ ^]^ 5,^[^ ^227^ ^]^ 6,^[^ ^350^ ^]^ 7,^[^ ^213^ ^]^ 8,^[^ ^40^ ^]^ 9^[^ ^351^ ^]^).

Optical modulators that operate due to the change of materials’ complex refractive indices play a significant role in integrated photonic circuits. In Si‐integrated photonic circuits, electric field (*E*) and power (*P*) of the optical signal that propagate in a waveguide along *z*‐direction changes exponentially as

(3) $$\vec{E}(z)={\vec{E}}_{0}\exp(-jk_{0}\cdot n_{\text{eff}}\cdot z-\alpha_{z}\cdot z)$$

(4) $$P(z)=P_{0}\cdot \exp(-\alpha_{z}\cdot z)$$

where ${\vec{E}}_{0}$ and $P_{0}$ are the electric field and power at *z* = 0, $k_{0}$ is the wavenumber, $\alpha_{z}$ is the absorption coefficient, and $n_{\text{eff}}$ is the effective refractive index. Refractive index and absorption coefficient are affected by carrier concentration (Si), which can be given by^[^ ^191^ ^]^

(5) $$n(\lambda )=-3.64\cdot 10^{-10}\cdot \lambda^{2}\cdot N-3.51\cdot 10^{-6}\cdot \lambda^{2}\cdot P^{0.8}$$

(6) $$\alpha (\lambda )=3.52\cdot 10^{-6}\cdot \lambda^{2}\cdot N+2.4\cdot 10^{-6}\cdot \lambda^{2}\cdot P[{\text{cm}}^{-1}]$$

where n and α are refractive index variation and absorption coefficient variation, P and N are holes and electrons concentration, and *λ* is the wavelength (m) of the incident light. From these two formulas, the output optical signal can be modulated by tuning *n* and *α* in Si‐integrated photonic circuits. According to different external fields that tune the complex refractive index of optoelectronic materials, optical modulators can be classified into all‐optical (AO), electro‐optic (EO), acousto‐optic, TO, mechano‐optic modulators, etc. Among these external fields, electric, optical, and thermal fields have been used for modulators based on 2DMs. Electric and optical fields have an impact on both the imaginary and real parts of the refractive indices, which can be tuned by the carrier concentration and nonlinear optical effects of 2DMs (**Figure** **6**).^[^ ^192^ ^]^ Generally, the thermal energy generated from 2DMs in a TO modulator can lead to the change of the refractive index of Si waveguide, which can reconfigure the optical light path or modulate the light intensity. However, heat dissipation is a relatively slow process; thus, the response time of TO modulators is usually in the range of microseconds. EO and electro‐absorptive (EA) modulators are based on the tunable complex refractive index induced by the change of carrier concentration and energy band. The switching time of EO and EA modulators is limited by the resistor–capacitor (RC) delay which is typically in the range from nanoseconds to a few picoseconds. In addition, taking advantage of the ultrafast saturable absorption, AO modulators can achieve a response time ranging from picoseconds to femtoseconds.

**Figure 7:**
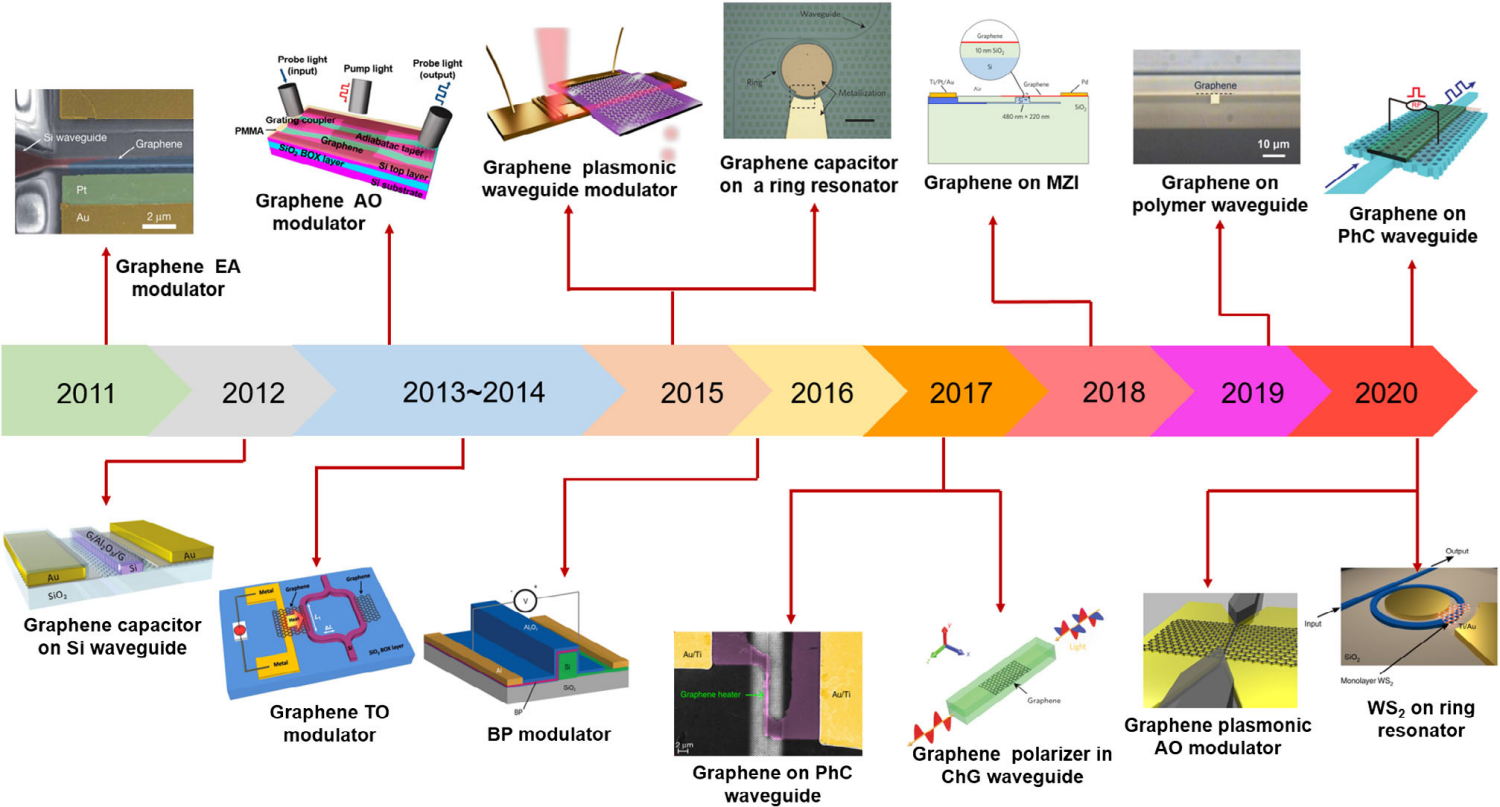
The development of a part of waveguide‐integrated modulators based on 2DMs. Graphene EA modulator (Reproduced with permission.^[^ ^37^ ^]^ Copyright 2011, Springer Nature). Graphene capacitor on Si waveguide (Reproduced with permission.^[^ ^223^ ^]^ Copyright 2012, American Chemical Society). Graphene AO modulator (Reproduced with permission.^[^ ^254^ ^]^ Copyright 2014, American Chemical Society). Graphene TO modulator (Reproduced with permission.^[^ ^352^ ^]^ Copyright 2014, AIP Publishing). Graphene plasmonic waveguide modulator (Reproduced under the terms of the CC‐BY 4.0 license.^[^ ^353^ ^]^ Copyright 2015, The Authors, published by Springer Nature). Graphene capacitor on a ring resonator (Reproduced with permission.^[^ ^227^ ^]^ Copyright 2015, Springer Nature). BP modulator (Reproduced with permission.^[^ ^240^ ^]^ Copyright 2016, American Chemical Society). Graphene on PhCW waveguide (Reproduced under the terms of the CC‐BY 4.0 license.^[^ ^220^ ^]^ Copyright 2017, The Authors, published by Springer Nature). Graphene polarizer in ChG waveguide (Reproduced with permission.^[^ ^43^ ^]^ Copyright 2017, Springer Nature). Graphene on MZI (Reproduced with permission.^[^ ^198^ ^]^ Copyright 2017, Springer Nature). Graphene on polymer waveguide (Reproduced with permission.^[^ ^354^ ^]^ Copyright 2019, Optical Society of America). Graphene plasmonic AO modulator (Reproduced with permission.^[^ ^40^ ^]^ Copyright 2019, Springer Nature). WS_2_ on ring resonator (Reproduced with permission.^[^ ^41^ ^]^ Copyright 2020, Springer Nature). Graphene on PhC waveguide (Reproduced under the terms of the CC‐BY 4.0 license.^[^ ^230^ ^]^ Copyright 2020, The Authors, published by de Gruyter).

Optical modulators based on 2DMs have attracted much attention because of their unique properties such as nonlinear optical response, broadband operation, and ultrafast carrier dynamics.^[^ ^193^, ^194^, ^195^, ^196^, ^197^ ^]^ In the past few years, optical modulators based on 2DMs have been an extensive research topic and great progress has been made.^[^ ^198^, ^199^, ^200^, ^201^, ^202^ ^]^ Specifically, 2DMs optical modulators such as mode‐locked fiber lasers,^[^ ^203^, ^204^ ^]^ Q‐switching lasers,^[^ ^205^, ^206^ ^]^ saturable absorbers,^[^ ^207^, ^208^ ^]^ polarization controllers,^[^ ^209^ ^]^ and wavelength converters^[^ ^210^ ^]^ have already been demonstrated. For example, graphene mode‐locked fiber lasers that generated pulse light with an ultrashort duration were achieved. In this passive self‐amplitude modulator, a hybrid structure of graphene and polymer was assembled between two fiber connectors with a fiber adaptor, which worked at the wavelength of 1559 nm, with a spectral bandwidth and pulse duration of 5.24 nm and ≈460 fs, respectively.^[^ ^211^ ^]^ Replacing graphene with TMDCs, a visible fiber laser was modulated.^[^ ^212^ ^]^ In addition, AO phase shifters were proposed by coating graphene,^[^ ^213^ ^]^ few‐layer WS_2_^[^ ^214^ ^]^ or phosphorene^[^ ^215^ ^]^ onto an optical fiber. Indeed, those optical modulators based on the optical fiber are not compatible with CMOS technology and it is difficult to realize on‐chip integrated photonic applications with these; thereby, waveguide‐integrated optical modulators are necessary. Up to now, most of the waveguide‐integrated 2DMs optical modulators are based on graphene (**Figure** **7**), which may be partially attributed to the fact that density of states near the Dirac point is ultralow and Fermi level (*E*_F_) can be easily tuned. In the following sections, the development of waveguide‐integrated optical modulators based on 2DMs will be introduced in detail according to different modulation mechanisms.

### 4.1 Thermo‐Optic Modulator

TO effect is the thermal modulation of the refractive index of optoelectronic materials. 2DMs play a significant role in generating the thermal energy in a TO modulator, in which the generated heating power can be expressed as

(7) $$P_{\text{heating}}=I^{2}\cdot R_{\text{tot}}$$

where $R_{\text{tot}}$ is the total resistance including resistance of 2DMs and contact resistance. Refractive index variation can be induced by the temperature change, leading to an optical phase shift (φ=2π·(L/λ)·(dn/dT)·ΔT), where d*n*/d*T* (TO coefficient) of Si is about 1.86 × 10^−4^ K^−1^ of at 1550 nm. So far, metal wires working as heaters are the primary material to control the temperature in the waveguide‐integrated TO modulator, in which a SiO_2_ gap is needed between the heater and waveguide to decrease parasitic loss due to strong light–metal interaction. Limited by the low thermal conductivity of SiO_2_ (1.44 W mK^−1^),^[^ ^216^ ^]^ heat conduction and dissipation can be hindered. In that case, 2DMs such as graphene working as the transparent heaters can be a better material choice for the TO modulator. Monolayer graphene possesses high thermal conductivity ranging from 4840 to 5300 W mK^−1^,^[^ ^217^ ^]^ which is one magnitude higher than Cu, and thereby it is suitable for thermal applications such as heaters and heat conductors. Although thermal diffusivity is not fast, limiting the modulation speed of the TO modulator, the modification of the refractive index is usually much higher than the carrier dispersion effect and the Pockels effect, leading to devices with a high extinction ratio (ER) and modulation depth (MD), low insertion loss, and small footprint.

**Figure 8:**
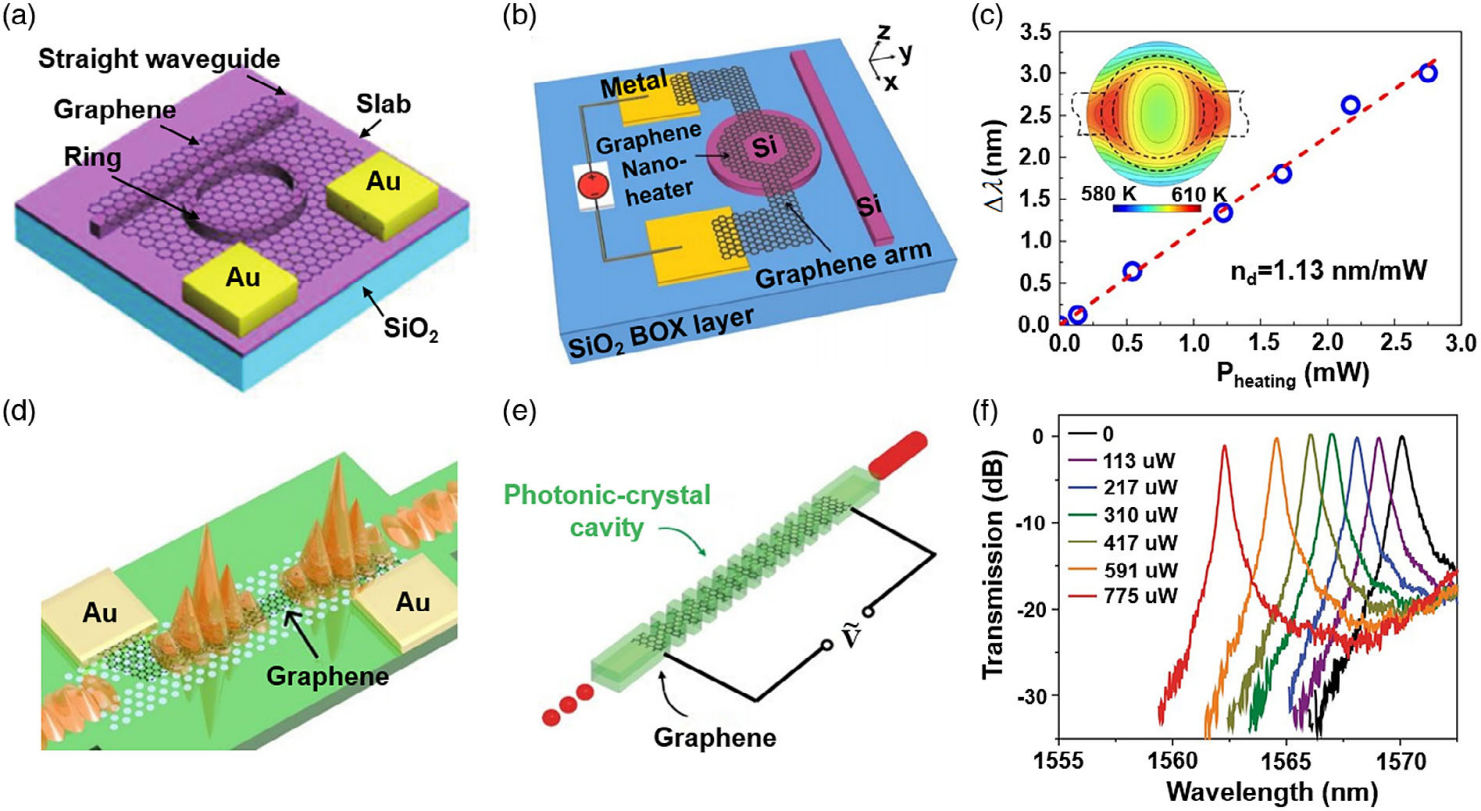
Waveguide‐integrated TO modulators. a) The illustration of a TO microring modulator. Reproduced with permission.^[^ ^218^ ^]^ Copyright 2015, The Royal Society of Chemistry. b) The schematic diagram of a microdisk modulator thermally tunable by a graphene nanoheater. c) The shift of the resonant wavelength under different heating energies. b,c) Reproduced with permission.^[^ ^219^ ^]^ Copyright 2016, Optical Society of America. d) The schematic diagram of the slow light‐enhanced graphene TO modulator. Reproduced under the terms of the CC‐BY 4.0 license.^[^ ^220^ ^]^ Copyright 2017, The Authors, published by Springer Nature. e) A TO switch with graphene integrated with a photonic‐crystal cavity. f) The transmission spectra of the waveguide‐integrated graphene optical switch under various input powers. e,f) Reproduced with permission.^[^ ^43^ ^]^ Copyright 2017, Springer Nature.

There are three common structures for TO modulators based on 2DMs up to now. First, a waveguide‐integrated resonator is a typical structure, partially resulting from the tunable resonant condition of the resonator with temperature. For example, a graphene heater directly coupled with a Si microring resonator has been designed for a TO modulator (**Figure** **8**a), in which the resonant wavelength shifts under different driving voltage.^[^ ^218^ ^]^ In this modulator, excess graphene on the straight waveguide generates an additional optical loss, leading to a large insertion loss. The optimization in device design for high‐*Q* cavities and efficient heaters is quite necessary to enhance the performance of TO modulators based on a resonator. For instance, a TO modulator based on a graphene heater on the microdisk resonator was designed (Figure 8b), and the higher heating efficiency was obtained, resulting from an optimal resistance of the nanoheater on the microdisk resonator. The temperature change induced by the graphene heater modified the resonant condition of the microdisk resonator (Figure 8c).^[^ ^219^ ^]^ Second, Mach–Zehnder interferometer (MZI) is another structure for TO modulators, and the photonic‐crystal waveguide was designed in this unbalanced MZI (Figure 8d).^[^ ^220^ ^]^ Compared with the free‐standing waveguide, this slow‐light photonic‐crystal waveguide had a large group index, which can boost phase accumulation in a short waveguide; thereby, power consumption per free spectral range of 3.99 mW and response time of 525 ns were realized. In addition, TO modulator has been achieved by the photonic‐crystal cavity in which a graphene heater was placed in the middle of the waveguide (Figure 8e).^[^ ^43^ ^]^ Figure 8f shows the transmission spectra of the waveguide‐integrated graphene optical switch under various input powers.^[^ ^43^ ^]^ Moreover, the energy efficiency of this device was up to 10 nm mW^−1^, which was the highest value reported. Such a high performance mainly results from the large spatial overlap between the heating zone and cavity mode as well as the strong thermal confinement. Actually, graphene possesses optical absorption in the telecommunication band, leading to degrading the optical modulation amplitude. Therefore, other 2DMs with a broad bandgap and high thermal conductivity may provide a possibility to realize a high‐performance TO modulators as well.

### 4.2 EA Modulator

**Figure 9:**
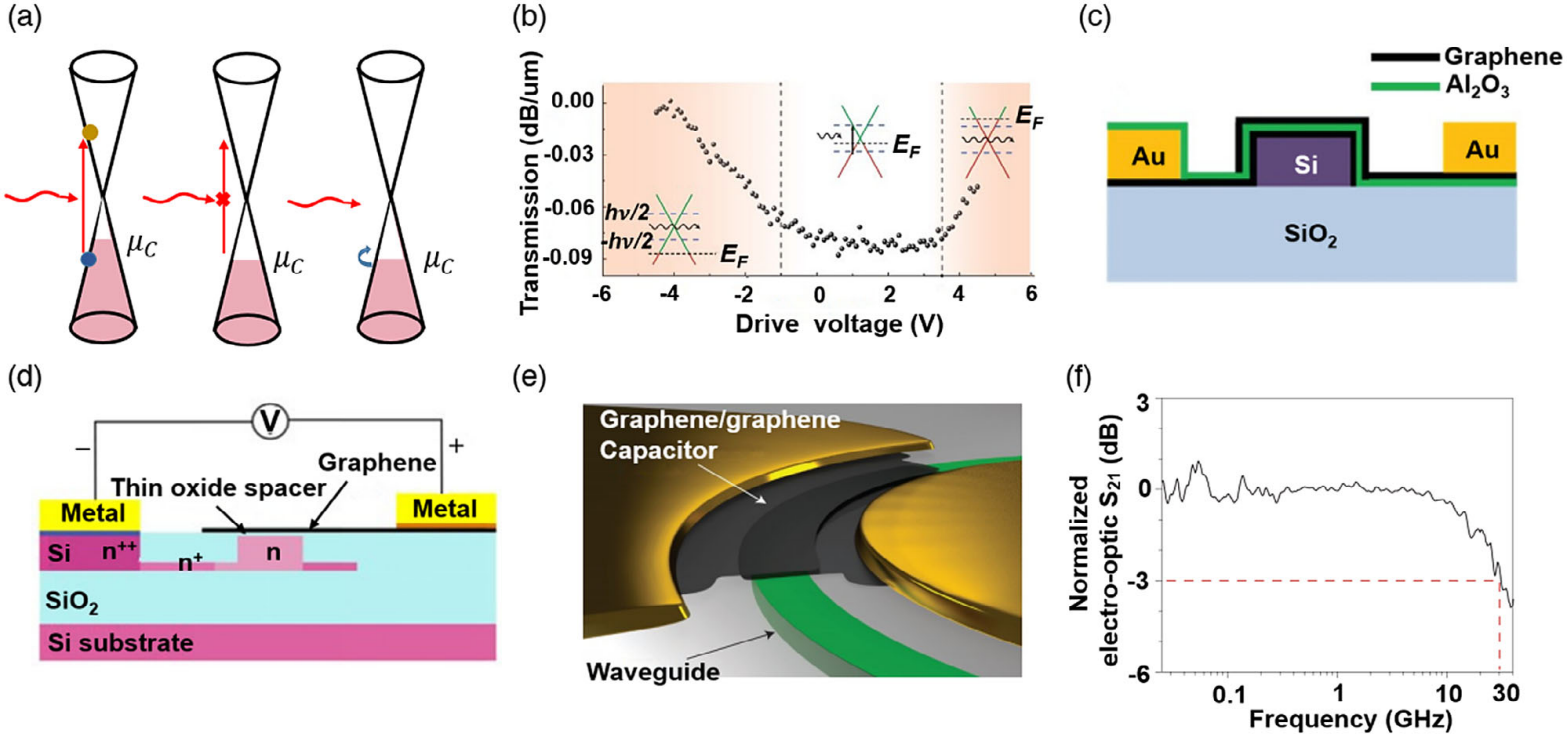
Waveguide‐integrated EA modulators. a) Interband and intraband optical absorption of graphene, $\mu_{c}$ is the chemical potential of graphene. b) The transmission curve of the graphene modulator under different external drive voltages. Reproduced with permission.^[^ ^37^ ^]^ Copyright 2011, Springer Nature. c) The schematic diagram of a double‐layer graphene modulator. Reproduced with permission.^[^ ^223^ ^]^ Copyright 2012, American Chemical Society. d) The schematic diagram of the novel EA modulator with doped silicon waveguide as gate electrode. Reproduced with permission.^[^ ^225^ ^]^ Copyright 2016, Wiley‐VCH. e) The illustration of the waveguide‐integrated graphene modulator coupled with a ring resonator. f) The measured frequency response of this ring‐resonator graphene modulator. e,f) Reproduced with permission.^[^ ^227^ ^]^ Copyright 2015, Springer Nature.

EA modulators can tune the intensity of light via an electric voltage. Graphene, with a Dirac cone‐shaped band structure, tunable property, and broadband optical absorption, is promising for the EA modulator. As shown in **Figure** **9**a, interband and intraband optical absorption are the main ways of graphene's optical absorption. Intraband optical absorption requiring a phonon‐assisted process is usually excited by a low‐energy photon such as MIR. Profound understanding of this process would promote developing the MIR modulator. At telecom wavelengths, interband absorption plays an important role. Electron transition from the valence band to the conduction band occurs under the excitation of photons whose energy is greater than 2$\mu_{c}$. In a capacitor, carrier concentration can be changed with charge accumulation and depletion, leading to the change of *E*_F_ as^[^ ^221^ ^]^

(8) $$E_{F}=\mu_{c}=\text{sgn}(n_{s})\hbar \upsilon_{F}\sqrt{\pi |n_{s}|}$$

where $n_{s}$ is the graphene surface carrier density, and $\nu_{F}$ is the Fermi velocity. Carrier change and *E*_F_ shift are affected directly by applied voltage (*V*)^[^ ^222^ ^]^

(9) $$|V-V_{\text{Dirac}}|=\frac{q\cdot n_{s}}{C_{\text{ox}}}+\frac{\left|\mu_{c}\right|}{q}=\frac{q}{C_{\text{ox}}}\frac{\mu_{c}^{2}}{\pi {\left(\hbar v_{F}\right)}^{2}}+\frac{\left|\mu_{c}\right|}{q}$$

where $V_{\text{Dirac}}$ is the flat‐band voltage depending on charge‐neutral Dirac point, $C_{\text{ox}}$ is the oxide capacitance per unit area. Usually, CVD graphene is lightly p‐doped and possesses an almost fixed charge‐neutral Dirac point. Therefore, the oxide capacitance per unit area and applied voltage are decisive factors to adjust $E_{F}$ of graphene.

The first graphene EA modulator was reported in 2011, in which CVD graphene was coated on a Si waveguide, and Al_2_O_3_ layer (7 nm) working as the dielectric layer was between graphene and Si waveguide.^[^ ^37^ ^]^ As shown in Figure 9b, graphene was transparent when |$\mu_{c}$| > ℏυ/2 and it was a light active material when |$\mu_{c}$| < ℏυ/2; thereby, an EA modulator (graphene–Al_2_O_3_–Si) was realized by tuning graphene's *E*_F_. The operational wavelength of this device ranged from 1.35 to 1.60 μm, covering *E* to *L* band in telecommunication. Graphene–Al_2_O_3_–graphene was another structure for the EA modulator. As shown in Figure 9c, one layer of graphene was doped by injected holes (electrons), and the other graphene layer was doped by electrons (holes).^[^ ^223^ ^]^ The ridge waveguide is necessary for the former modulator (graphene–insulator–Si), and a heavily doped process is necessary to ensure Ohmic contact between Si and electrode. In contrast, both strip waveguide and ridge waveguide are suitable for the latter modulator (graphene–insulator–graphene), and a heavily doped process can be avoided, which reduces fabrication cost. Moreover, RC‐limited bandwidth in such a device depends on the series resistance and capacitance as^[^ ^224^ ^]^

(10) $$f_{3\text{dB}}=\frac{1}{2\cdot \pi \cdot R_{s}\cdot C_{m}}$$

where $R_{s}$ is the series resistance, and $C_{m}$ is the total capacitor. $C_{m}$ contains series connection of quantum capacitance ($C_{Q}$) of each layer of graphene and Al_2_O_3_ capacitance per unit area ($C_{A}$), which can be expressed as^[^ ^224^ ^]^

(11) $$C_{m}=\frac{0.5\cdot C_{Q}\cdot C_{A}}{C_{A}+0.5\cdot C_{Q}}\cdot W_{m}\cdot L_{m}$$

where $W_{m}$ and $L_{m}$ are the width and length of modulator. In a practical modulator, series resistance is mainly attributed to the sum of contact resistance ($R_{C}$) and sheet resistance ($R_{\text{sh}}$); thereby, $f_{3\text{dB}}$ can be expressed approximately as^[^ ^224^ ^]^

(12) $$f_{3\text{dB}}\approx \frac{1}{2\cdot \pi \cdot (R_{C}+R_{\text{sh}}\left(W_{m}+W_{e}\right)\cdot L_{m}}\cdot \frac{2\cdot C_{A}+C_{Q}}{C_{A}\cdot C_{Q}\cdot W_{m}\cdot L_{m}}$$

where $W_{e}$ is the sum width between electrode and waveguide edge.

Reducing resistance and capacitance of devices brings about high‐performance modulators. An effective approach was proposed to reduce device resistance while maintaining low‐free‐carrier absorption from silicon waveguide by controlling different doping levels of Si at different locations (Figure 9d). Benefitting from this optimal structure, therefore, modulation speed reached up to 10 Gb s^−1^ at the wavelength from 1530 to 1565 nm.^[^ ^225^ ^]^ Recently, a theoretical investigation showed an EA modulator based on the suspended self‐biasing graphene waveguide, which could not only increase light–matter interaction but also decreased resistance and capacity of the device because of strong light confinement in graphene.^[^ ^226^ ^]^ Although a faster speed can be obtained with a thicker gate oxide, a lower capacitance results in the weaker gating effect and weaker capability to tune the carrier concentration in graphene. The trade‐off between response time and modulation efficiency has been overcome by integrating with a microring resonator (Figure 9e).^[^ ^227^ ^]^ As the transmission at resonance is susceptible to the optical loss within the microring, the modulation area for the micro‐ring modulator could be kept with a small footprint whereas the MD could maintain a very high on/off ratio. Thus the fabricated devices could achieve a 3 dB bandwidth as high as 30 GHz (Figure 9f).

**Figure 10:**
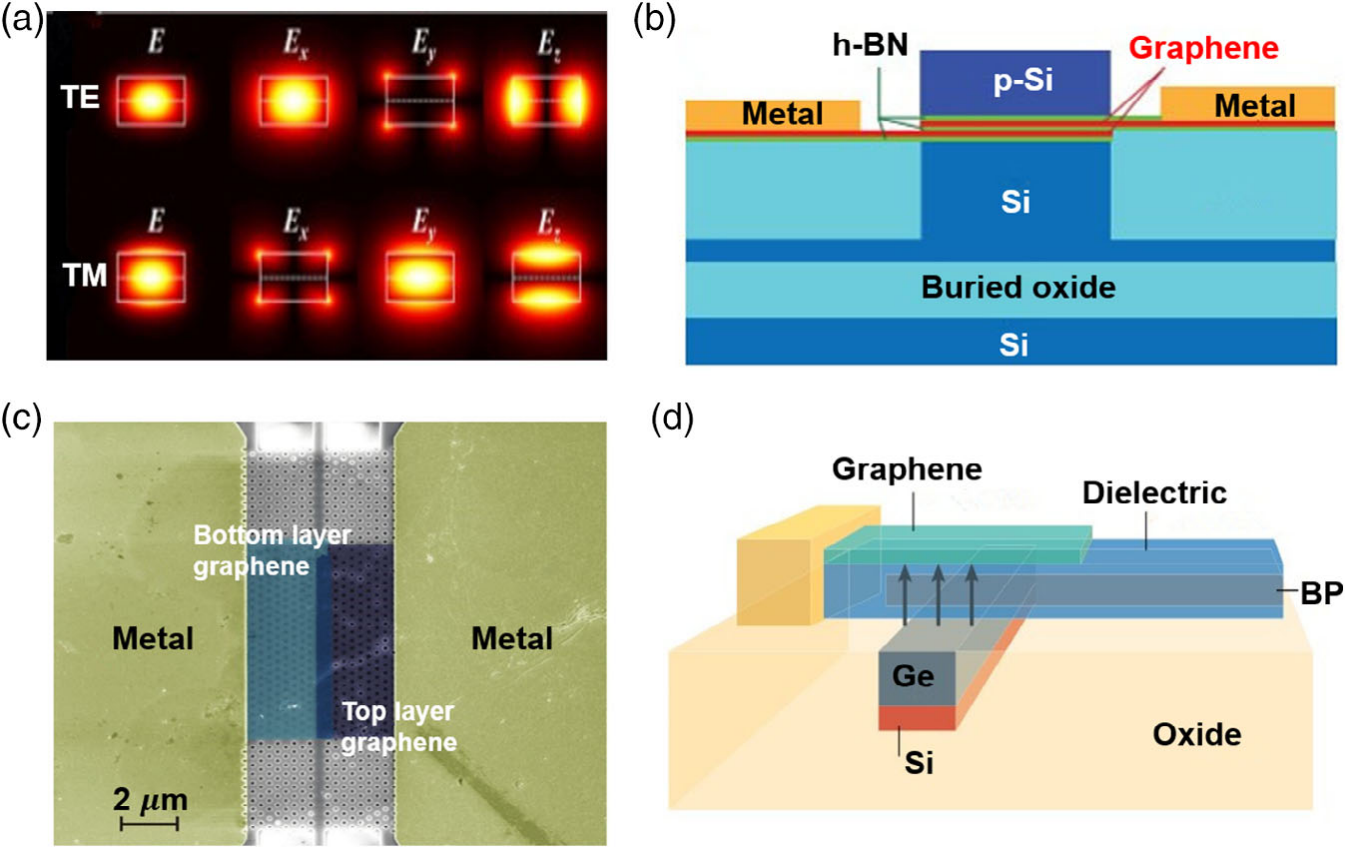
Waveguide‐integrated EA modulator. a) Simulated field distribution for TE and TM modes in a waveguide–graphene–waveguide structure. Reproduced with permission.^[^ ^43^ ^]^ Copyright 2017, Springer Nature. b) A novel modulator where graphene is located in the middle of waveguide. Reproduced with permission.^[^ ^137^ ^]^ Copyright 2011, Springer Nature. c) Graphene‐based modulator on a photonic‐crystal waveguide. Reproduced under the terms of the CC‐BY 4.0 license.^[^ ^230^ ^]^ Copyright 2020, The Authors, published by de Gruyter. d) MIR modulator based on BP and Ge waveguide. Reproduced with permission.^[^ ^241^ ^]^ Copyright 2019, Springer Nature.

Enhancing optical absorption is another practical approach to improve the performance of EA modulators, especially for low power consumption, a high ER, and MD, which are expressed as

(13) $$\text{ER}=10\cdot \text{log}(P_{\text{max}}-P_{\text{min}})$$

(14) $$\text{MD}=10\cdot \text{log}\frac{P_{\text{max}}-P_{\text{min}}}{P_{\text{max}}}$$

where $P_{\text{max}}$ and $P_{\text{min}}$ are maximal and minimal output power of modulator, which are influenced by the absorption coefficient, as shown in Equation (4). Usually, a larger absorption coefficient brings about a device with a smaller volume, and thereby it can reduce resistance and improve response speed as well. First, numerical analysis indicated that light intensity is maximum in the middle of a waveguide (**Figure** **10**a); thereby, placing optical materials in this region can enhance the light–matter interaction. Based on this device configuration (Figure 10b), both MD and operational speed could be improved, and the intrinsic bandwidth of the graphene modulator was predicted to be 55 GHz.^[^ ^137^ ^]^ Although this is an intriguing phenomenon to enhance optical absorption, it's challenging to fabricate such a device in the SOI platform as we mentioned in the introduction. The optical waveguide made from new materials such as ChG possesses obvious advantages for fabrication.^[^ ^47^, ^228^, ^229^ ^]^ Second, the photonic‐crystal waveguide is a typically used structure for improving the performance of the EA modulator because of the slow light effect. Integrating graphene with the Si photonic‐crystal waveguide, high‐performance EA modulators can be obtained (Figure 10c).^[^ ^230^ ^]^ Third, surface plasmon induced by the collective oscillation of electrons is another light‐trapping structure that can enhance light–matter interaction,^[^ ^231^, ^232^, ^233^ ^]^ which has been widely used to improve the performance of EA modulators in the past few years.^[^ ^234^, ^235^ ^]^ For instance, Ag nanocylinders were coated onto graphene on the Si waveguide; the numerical investigation indicated that MD and modulation speed were 4 dB μm^−1^ (2.6 dB loss) and 39 GHz, respectively.^[^ ^236^ ^]^ The surface plasmon is an effective way to improve optical absorption; thereby, the device length has to be minimized to reduce the large insertion loss. In addition, patterning graphene,^[^ ^237^ ^]^ adding reflective structure,^[^ ^66^ ^]^ taking advantage of interference improvement,^[^ ^238^ ^]^ and coupling with evanescent mode^[^ ^239^ ^]^ are possible approaches to enhance light–matter interaction.

Besides graphene, other 2DMs have also shown unique advantages for EA modulators as well. A theoretical study demonstrated that a waveguide‐integrated EA modulator based on multilayer BP could play a significant role in optical modulation in the MIR region.^[^ ^240^ ^]^ The optical bandgap of BP can be tunable with an external gate voltage because of the quantum‐confined Stark effect and Burstein–Moss shift. Integrating BP with germanium waveguide (Figure 10d),^[^ ^241^ ^]^ a modulator could be designed to operate at a broadband ranging from 4 to 12 μm, thanks to the large tuning range of bandgap energy of BP sandwiched between the dielectric layers, which could be tuned from 0.33 eV to 0.075 eV.^[^ ^242^ ^]^ In addition, free‐space EA modulators based on multilayer BP have also been demonstrated.^[^ ^210^, ^243^ ^]^ Although the operational wavelength of modulators has been extended to the MIR region, high‐performance waveguide‐integrated EA modulators based on BP have rarely been achieved.

### 4.3 EO Modulator

**Figure 11:**
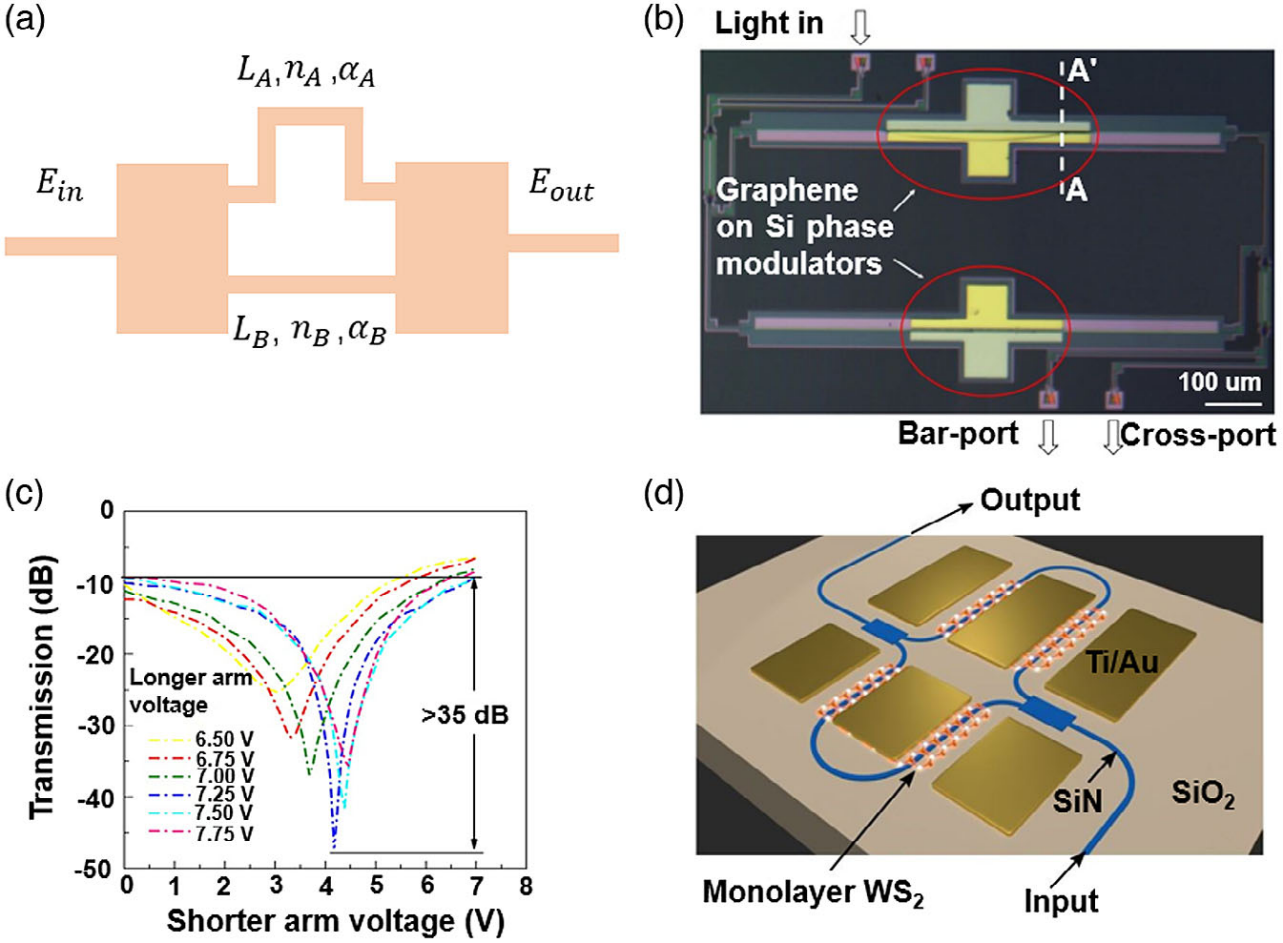
Waveguide‐integrated EO modulators. a) Schematic diagram of unbalanced MZI. b) The illustration of graphene EO phase modulator based on unbalanced MZI. c) The ER (1550 nm) of graphene MZI phase modulator under different applied voltages. b,c) Reproduced with permission.^[^ ^198^ ^]^ Copyright 2017, Springer Nature. d) An EO phase modulator based on WS_2_. Reproduced with permission.^[^ ^41^ ^]^ Copyright 2020, Springer Nature.

Compared with the EA modulator, the EO modulator is mainly based on tuning the real part of the refractive index induced by external electric fields. Thus the phase, amplitude, and polarization of the light can be modulated. Most of EO modulators are based on the MZI that consists of a couple of multimode interferometers (MMI) and waveguides. As shown in **Figure** **11**a, we assume that the left MMI divides input power into two equal parts without phase difference, the electric fields in two arms in the input of the right MMI can be expressed as

(15) $$E_{A}^{\text{′}}=\sqrt{0.5\cdot \alpha \cdot P_{\text{in}}}\cdot \text{exp}(-jk_{0}n_{A}L_{A}-\alpha_{A}L_{A})$$

(16) $$E_{B}^{'}=\sqrt{0.5\cdot \alpha \cdot P_{\text{in}}}\cdot \text{exp}(-jk_{0}n_{B}L_{B}-\alpha_{B}L_{B})$$

where *α* is the loss factor of the left MMI, $P_{\text{in}}$ is the input power, $n_{A}$ ($n_{B}$), $L_{A}$ ($L_{B}$), $\text{and}\alpha_{A}$ ($\alpha_{B}$) are refractive index, length, and optical absorption of arm A (B), respectively. Thus, the output power can be expressed as

(17) $$P_{\text{out}}=\alpha \cdot \beta \cdot P_{\text{in}}\cdot \{0.5\cdot \exp(-2\alpha_{A}\cdot L_{A})+0.5\cdot \exp(-2\alpha_{B}\cdot L_{B})+\exp(-\alpha_{A}\cdot L_{A}-\alpha_{B}\cdot L_{B})\cdot \cos[k_{0}\cdot (n_{B}\cdot L_{B}-n_{A}\cdot L_{A})]\}$$

where *β* is the loss factor of the right MMI. According to this equation, the output power is maximal when $n_{B}\cdot L_{B}-n_{A}\cdot L_{A}=0$ or 2kπ (*k* is an integer), whereas it is minimal when $n_{B}\cdot L_{B}-n_{A}\cdot L_{A}=2k\pi -1$. Phase modulator has already been realized by the graphene and Si spiral waveguide hybrid platform, in which *π*/3 phase shift has be achieved with a device area of 32.252 μm^2^ under the applied voltage of 1.1–2.3 V.^[^ ^244^ ^]^ Recently, a compact and high‐speed waveguide‐integrated graphene phase modulator integrating with MZI was reported (Figure 11b).^[^ ^198^ ^]^ The basic structure of this device was a graphene–insulator–silicon capacitor, which was similar to the modulator based on the metal–oxide semiconductor capacitor in Si photonics. Carriers accumulate at the graphene–SiO_2_ and Si–SiO_2_ interface under a gate voltage, leading to the change of the refractive index and optical absorption coefficient, which is induced by the plasma dispersion effect as follows^[^ ^245^, ^246^ ^]^

(18) $$\text{Si}(1550\text{ nm}):\;\{\begin{array}{c} n=-5.4\cdot 10^{-22}\cdot N^{1.011}-1.53\cdot 10^{-18}\cdot P^{0.838}\\ \alpha =8.88\cdot 10^{-21}\cdot N^{1.167}+5.84\cdot 10^{-20}\cdot P^{1.109} \end{array}$$

(19) $$\text{Graphene}:\;\{\begin{array}{c} n=\sqrt{\epsilon \cdot \mu }\\ \epsilon =1+\frac{i\sigma \left(\omega \right)}{w\cdot \epsilon_{0}\cdot h} \end{array}$$

where *ϵ* and μ are the in‐plane dielectric constant and permeability of graphene, *h* is the thickness of graphene, *w* is the radian frequency, and σ(ω) is the complex surface conductivity of graphene. N, P, andσ(ω) are affected by an applied voltage. When applying 4.1 V to the shorter arm and 7.25 V to the longer arm, the phase difference of light signal between the two arms was *π*, leading to minimal output power (Figure 11c). This modulator possessed a modulation efficiency of 0.28 V cm at 1550 nm and a 3 dB bandwidth of 5 GHz with a phase‐shifter length of 300 μm. The modulation efficiency of this modulator is better than that observed in the conventional Si PN junction.^[^ ^247^, ^248^ ^]^ Si does not show Pockels effect [limited] by symmetric atom structure, and most of the EO modulators (Si) are based on the plasma dispersion effect. Combining Si with 2DMs, the fabrication for a 2DM–insulator–Si capacitor is simpler than that of a traditional Si modulator based on plasma dispersion effect as 2DM–insulator–Si capacitor avoids several different doping processes. Very recently, WS_2_ and MoS_2_ also showed their ability to realize the EO modulator based on the plasma dispersion effect (Figure 11d), which results in a modulator with a lower insertion loss and higher MD. Taking full advantage of 2DMs, a high‐performance phase modulator with increased modulation efficiency, reduced optical loss, and miniaturized footprint would be realized in the future, which is significant for integrated photonics.

### 4.4 AO Modulator

**Figure 12:**
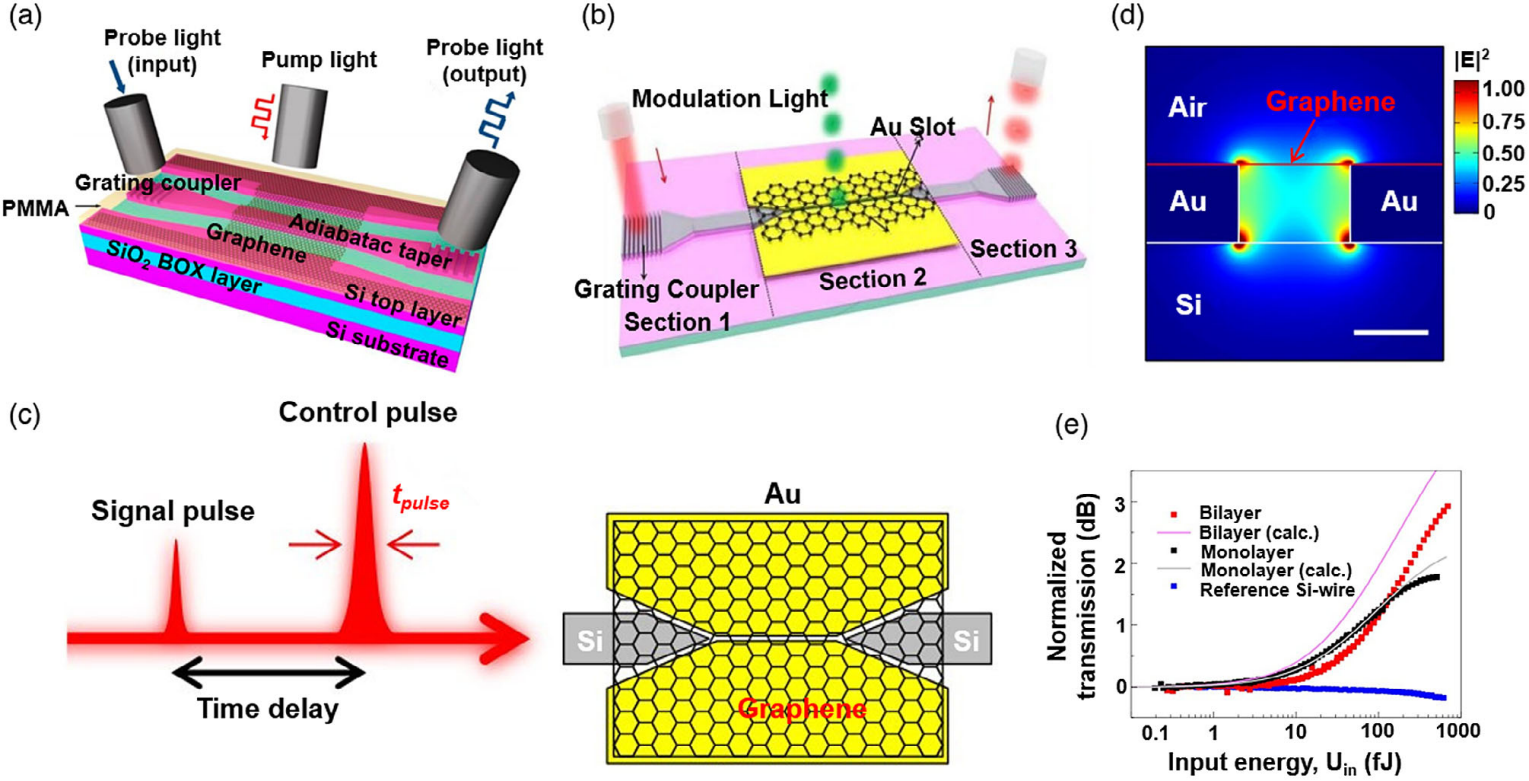
Waveguide‐integrated AO modulator. a) Illustration of waveguide‐integrated local and nonlocal AO graphene modulator. Reproduced with permission.^[^ ^254^ ^]^ Copyright 2014, American Chemical Society. b) Schematic of the graphene‐plasmonic slot waveguide AO modulator. Reproduced with permission.^[^ ^257^ ^]^ Copyright 2019, The Japan Society of Applied Physics. c) Schematic of the graphene‐plasmonic slot waveguide AO modulator and pump‐probe measurement. d) Calculated field profile (|*E*|^2^) of graphene AO modulator. e) Saturable absorption of the monolayer, bilayer graphene, and Si wire with excitation of picosecond laser pulses. c–e) Reproduced with permission.^[^ ^40^ ^]^ Copyright 2019, Springer Nature.

The AO modulator, avoiding conversion between optical and electrical signals, has been extensively studied in the past few years.^[^ ^249^, ^250^, ^251^ ^]^ It can not only eliminate the bandwidth limitation of traditional modulators but also meets the requirement of ultrafast operational speed and ultralow energy consumption, which may lead to the development of ultrafast optical communication and computation.^[^ ^252^ ^]^ Till now, several different mechanisms for the AO modulator based on graphene/waveguide have been demonstrated. On the one hand, *E*_F_ of graphene can be changed with the injection of photoinduced carriers from semiconductor waveguide to graphene; thereby, graphene extinction coefficient can be tunable.^[^ ^253^ ^]^ Based on this principle, the optically induced transparency effects were realized by graphene/Si nanophotonic integrated circuits (**Figure** **12**a), where pump power was ≈2 W cm^−2^, and dynamic responses in various positions were different.^[^ ^254^ ^]^ Although this type of modulator is excited by the optical signal, avoiding electrical‐to‐optical conversion, advantages of AO modulators such as fast speed are limited by the carrier transit time. Moreover, photogenerated carriers are excited by pump light at the wavelength of 532 nm in this Article. In integrated photonic circuits, however, pump light and probe light are supposed to be coupled into the same waveguide; thereby, the wavelength of pump light better be at the transparent window of Si as well. On the other hand, light absorbed by graphene or BP could generate heat energy which modifies the temperature and the refractive index of the waveguide.^[^ ^255^, ^256^ ^]^ The response speed of those modulators based on optical‐induced TO effect could be a few nanoseconds. Limited by the speed of carrier transfer and thermal dissipation, it is difficult to obtain an ultrafast response. Recently, plasmonic slot waveguide has shown its advantages in realizing a 2DM AO modulation because of local field enhancement induced by surface plasmon polariton (SPP). An AO modulator based on graphene plasmonic slot waveguide was proposed (Figure 12b) with the modulation efficiency of 0.21 dB μm^−1^.^[^ ^257^ ^]^ Once pump light is propagated along this plasmonic slot waveguide (Figure 12c), light–graphene interaction could be enhanced dramatically (Figure 12d). Benefiting from a small volume of light propagation mode and local field enhancement, the saturation energy of graphene is measured to be 12 fJ (Figure 12e), which is much smaller than graphene onto a Si slot waveguide. This AO modulation showed an ultrafast switching time of 260 fs due to the fast response speed of graphene saturable absorption,^[^ ^40^ ^]^ which indicated that saturable absorption could be applied for designing novel ultrafast, ultralow‐power AO modulators. Although a fast AO modulator can be realized by plasmonic slot waveguide, insertion loss is an inevitable disadvantage induced by metal absorption.

### 4.5 Conclusion

In the past decade, waveguide‐integrated optical modulators based on 2DMs have made a series of progress in both theoretical and experimental aspects. Figures of merit of state‐of‐the‐art waveguide‐integrated 2DM modulators are shown in **Table** **2**. Although these modulators based on 2DMs with high performance have shown great potential for integrated photonic circuits, there are some remaining challenges. First, most of the devices demonstrated today were operating at telecom wavelengths, extended optical modulation to the visible and MIR could pave for more fascinating applications for chemical/biosensing and visual reality. TMDCs and narrow‐bandgap MXene would be great material candidates. Second, pursuing lower power consumption and ultrafast modulation speed is a goal for integrated photonic circuits. Embedding 2DMs into the photonic structure and taking advantage of resonance or plasmonic enhancement could significantly reduce the device footprint. Thus the thermal mass or the parasitic capacitance could be minimized to achieve unprecedented low power consumption. Last but not least, more modulation approaches including acoustic, strain‐induced, ferroelectric modulation based on 2DMs are still waiting for exploration, which could provide new research opportunities in the near future.

**Table 2:** Figures of merit in typical waveguide‐integrated modulators based on 2DMs

Material and device structure	Type	Bandwidth	Footprint	MD	Insertion loss	Ref
Graphene on a silicon microring resonator	TO	0.23 MHz	10 μm^2^	7 dB	NR	[218]
Graphene on Si_3_N_4_ microring resonator	TO	1.38 MHz	3600 μm^2^	NR	NR	[256]
CVD graphene on Si waveguide with Al_2_O_3_ layer	EA	1.2 GHz	25 μm^2^	4 dB	NR	[37]
Double‐layer graphene on Si waveguide	EA	≈1 GHz	80 μm^2^	6.5 dB	≈4 dB	[223]
Si waveguide on double‐layer graphene	EA	35 GHz	18 μm^2^	2 dB	0.9 dB	[134]
Graphene on Si waveguide	EA	670 MHz	NR	16 dB	3.3 dB	[360]
Graphene–SiO_2_–Si waveguide	EA	5.9 GHz	500 μm^2^	5 dB	3.8 dB	[225]
Double graphene on a ring Si_3_N_4_ waveguide	EA	30 GHz	3600 μm^2^	22 dB	≈12.5 dB	[227]
Graphene on MZI	EO	5 GHz	NR	35 dB	NR	[198]
Graphene on microfiber	AO	159 GHz	NR	1.4 dB	NR	[213]
BP on a silicon microring resonator	AO	2.5 MHz	200 μm^2^	NR	NR	[255]
Graphene on plasmonic waveguide	AO	1.35 THz	600 nm^2^	NR	19 dB	[40]

## 5 Waveguide‐Integrated Photodetectors

**Figure 13:**
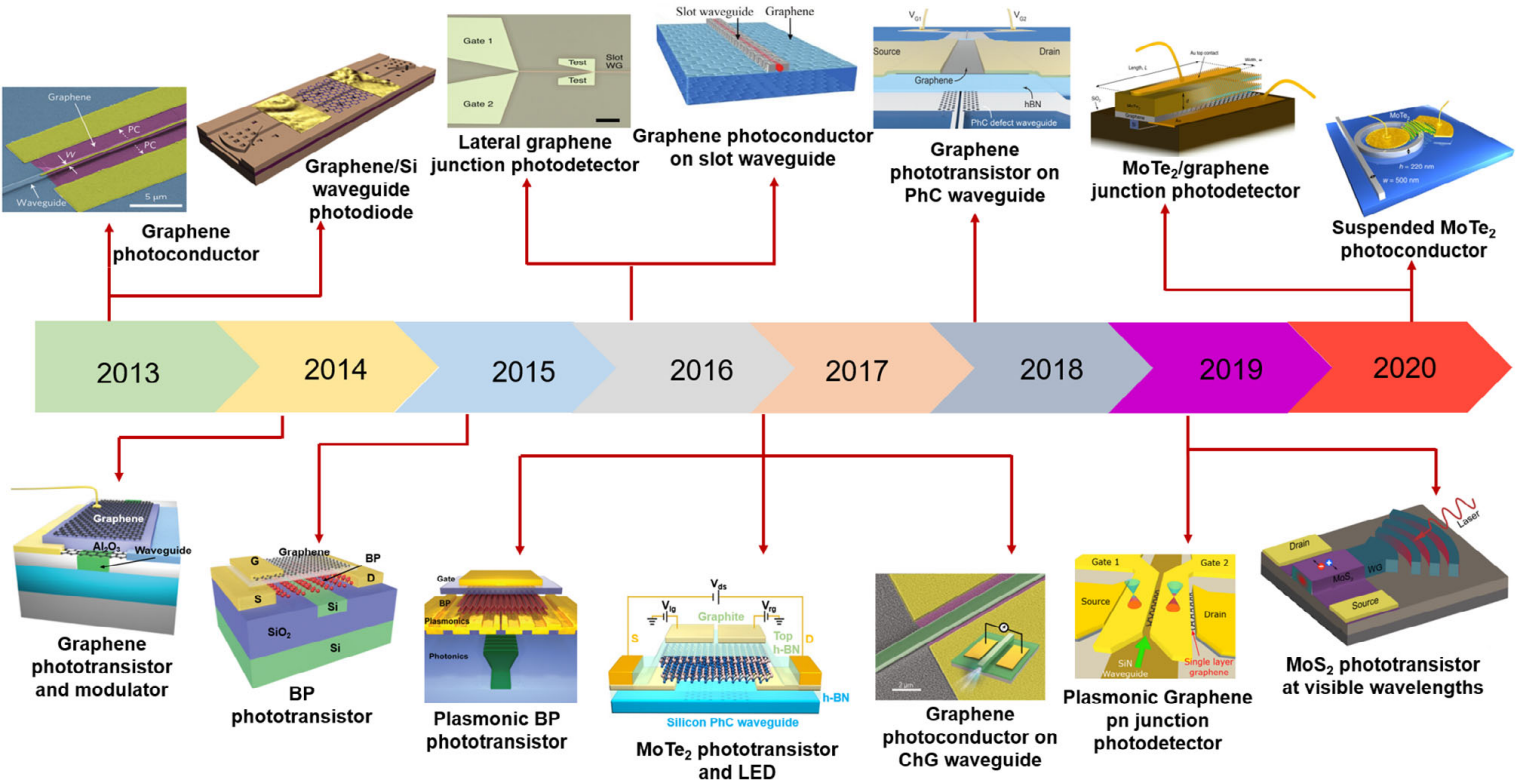
The development of a part of waveguide‐integrated photodetectors based on 2DMs. Graphene photoconductor (Reproduced with permission.^[^ ^355^ ^]^ Copyright 2013, Springer Nature). Graphene/Si waveguide photodiode (Reproduced with permission.^[^ ^356^ ^]^ Copyright 2013, Springer Nature). Graphene phototransistor and modulator (Reproduced with permission.^[^ ^296^ ^]^ Copyright 2014, American Chemical Society). BP phototransistor (Reproduced with permission.^[^ ^299^ ^]^ Copyright 2015, Springer Nature). Lateral graphene junction photodetector (Reproduced with permission.^[^ ^297^ ^]^ Copyright 2016, American Chemical Society). Graphene photoconductor on slot waveguide (Reproduced with permission.^[^ ^364^ ^]^ Copyright 2016, The Royal Society of Chemistry). Plasmonic BP phototransistor (Reproduced with permission.^[^ ^305^ ^]^ Copyright 2017, American Chemical Society). MoTe_2_ phototransistor and LED (Reproduced with permission.^[^ ^133^ ^]^ Copyright 2017, Springer Nature). Graphene photoconductor on ChG waveguide (Reproduced with permission.^[^ ^43^ ^]^ Copyright 2017, Springer Nature). Graphene phototransistor on PhC waveguide (Reproduced with permission.^[^ ^357^ ^]^ Copyright 2018, American Chemical Society). Plasmonic Graphene pn junction photodetector (Reproduced with permission.^[^ ^359^ ^]^ Copyright 2019, American Chemical Society). MoS_2_ phototransistor at visible wavelengths (Reproduced with permission under the terms of the CC‐BY 4.0 license.^[^ ^358^ ^]^ Copyright 2019, The Authors, published by Springer Nature). MoTe_2_/graphene junction photodetector (Reproduced with permission.^[^ ^42^ ^]^ Copyright 2020, Springer Nature). Suspended MoTe_2_ photoconductor (Reproduced with permission.^[^ ^289^ ^]^ Copyright 2020, Springer Nature).

In the past decade, free‐space photodetectors based on 2DMs have attracted a lot of attention^[^ ^258^, ^259^, ^260^, ^261^, ^262^, ^263^, ^264^, ^265^, ^266^, ^267^, ^268^ ^]^ and the related work has been summarized in several Review Articles.^[^ ^123^, ^269^, ^270^, ^271^, ^272^, ^273^ ^]^ As for integrated photonic circuits, waveguide‐integrated photodetectors based on 2DMs are vital components, which possess several specific advantages.^[^ ^99^ ^]^ First, the waveguide‐integrated design promises on‐chip detectors with a minimized dimension, enabling seamless planar integration with other photonic devices. Second, the signal‐to‐noise ratio (SNR) of photodetectors can be significantly improved, benefiting from the reduced detection volume. In addition, both the broad bandwidth and high quantum efficiency can be achieved simultaneously in waveguide‐integrated photodetectors, for the carrier collection path and light propagation direction are orthogonal, and there is no trade‐off between them. Up to now, lots of intriguing phenomena and optimal device structures have been proposed to design waveguide‐integrated photodetectors which are partially concluded in **Figure** **13**.

**Figure 14:**
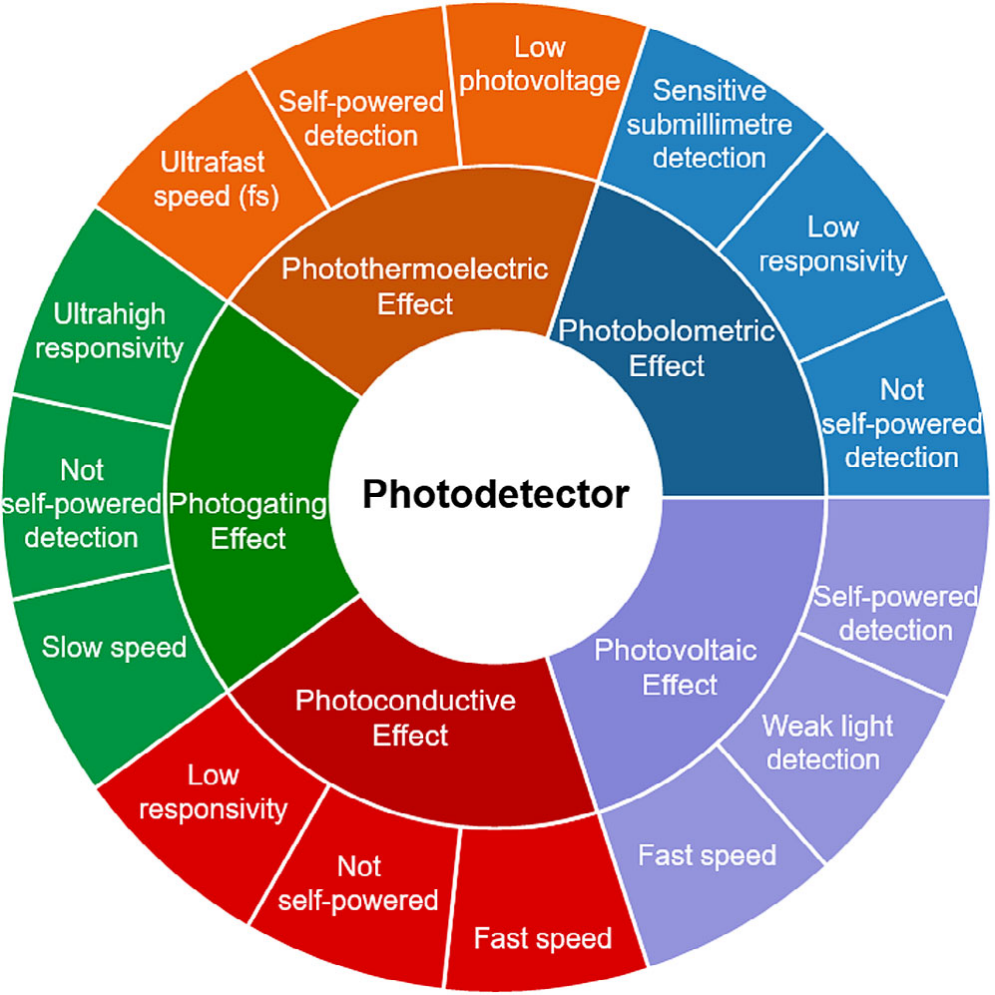
Characteristics of 2DM photodetectors based on different operational mechanisms that consist of the PV effect, photoconductive (PC) effect, photothermoelectric effect (PTE), photobolometric effect (PBE), and photogating effect (PG).

The operational mechanisms of the photodetectors are shown in **Figure** **14**. Among these five mechanisms, photodetectors based on the photovoltaic (PV) effect and PTE can work well without an external bias, which is, however, necessary for the operation of PC, PBE, and PE photodetectors. For PV photodetectors, a PN or heterojunction junction is necessary, in which the built‐in electric field drives the separation of photogenerated carriers, generating photovoltage and photocurrent without external bias. As for 2DM PV detectors, junctions can be constructed via homojunctions or heterojunctions, local chemical doping,^[^ ^274^ ^]^ as well as the modulation of the split‐gate electrode.^[^ ^275^ ^]^ PTE detectors could also operate without external bias, and photocurrent is directly related to the thermal gradient of 2DMs under light illumination. Besides graphene that has been used for high‐speed PTE photodetectors with broadband operation,^[^ ^276^, ^277^, ^278^, ^279^ ^]^ 2DMs such as MoS_2_, SnS_2_, and BP have also been proved to be promising candidates for thermoelectric photodetectors.^[^ ^280^, ^281^, ^282^ ^]^ The other types of photodetectors are based on measuring the change of conductance, whose mechanism could be classified into PBE, PC, and PG effects. Among these three effects, PBE results from the change of carrier mobility (*μ*) due to the local temperature variation, PC and PG arise from the changes in the number of carriers (*n*). Trap states inside 2DMs could generate a large gain for PC and PG photodetectors, a large gain would result in high responsivity but sacrificing the response speed. Usually, PTE and PBE photodetectors do not have gain.

The performance metrics characterizing photodetectors, such as operational wavelength, responsivity, noise equivalent power (NEP), and response speed, are directly related to the optoelectronic materials. The operational wavelength mainly depends on the bandgap of materials. As reported, 2DMs offer a wide range of bandgap selection, suitable for photodetectors working under various wavelengths. Specifically, wide‐bandgap 2DMs such as h‐BN are suitable for ultraviolet detection.^[^ ^283^ ^]^ TMDCs and 2D perovskites are preferred materials for visible light detection.^[^ ^284^ ^]^ When detection wavelength ranges from 1300 to 4000 nm, narrow‐bandgap semiconductors such as BP, As_1−*x*_P_*x*_, and PtSe_2_ can be ideal candidates.^[^ ^96^, ^285^, ^286^ ^]^ BP, presenting high carrier mobility over 1000 cm^2^ V^−1^ s^−1^ and a direct bandgap ranging from 0.3 to 2.1 eV with different layers,^[^ ^241^ ^]^ is an attractive 2DM for photodetectors. Graphene, being a gapless semimetal material, can be utilized for ultra‐broadband detection with spectral response from ultraviolet to terahertz,^[^ ^259^, ^287^, ^288^ ^]^ which is unrivaled by other 2DMs and is attractive for on‐chip optical communication in which data can be multiplexed and transmitted at a very high speed over a broad wavelength range.

**Figure 15:**
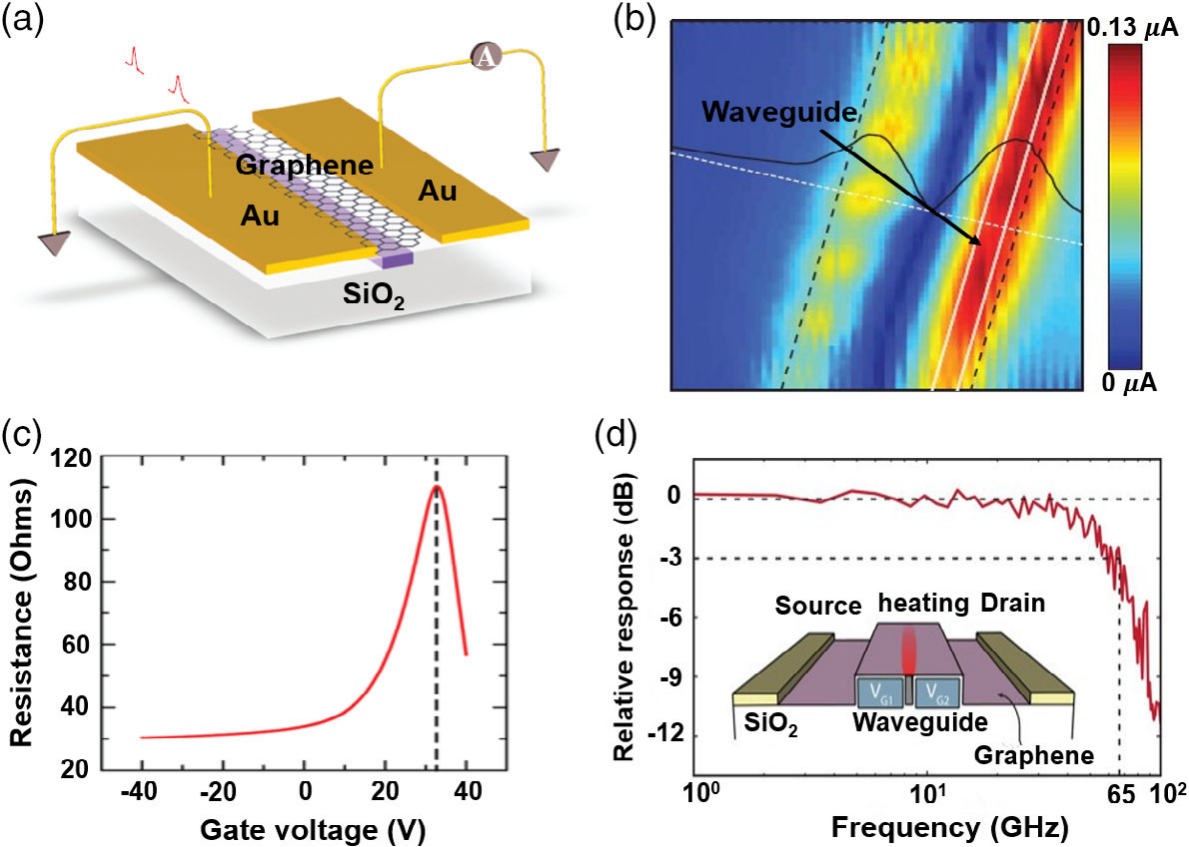
a) The schematic of waveguide‐integrated graphene photodetectors. b) Spatially resolved photocurrent mapping of the photodetector under zero external bias. a,b) Reproduced with permission.^[^ ^38^ ^]^ Copyright 2013, Springer Nature. c) The resistance variation of graphene under different gate voltages. Reproduced with permission.^[^ ^296^ ^]^ Copyright 2014, American Chemical Society. d) The schematic and photoresponse of the gate‐controlled graphene PN junction photodetector. Reproduced with permission.^[^ ^297^ ^]^ Copyright 2016, American Chemical Society.

Benefitting from ultrafast carrier mobility and optical absorption in a wide energy spectrum, graphene was the first 2DM to be applied for waveguide‐integrated photodetection. Usually, the total bandwidth is influenced by RC response and carrier transit time^[^ ^191^ ^]^

(20) $$f_{3\text{dB}}={\left(\frac{1}{f_{\text{RC}}^{2}}+\frac{1}{f_{\text{transit}}^{2}}\right)}^{-1/2}$$

where $f_{\text{RC}}$ and $f_{\text{transit}}$ are the bandwidth limited by RC and transit time limitations, respectively. The transit time ($\tau_{\text{transit}}$) is given by^[^ ^289^ ^]^

(21) $$\tau_{\text{transit}}=\frac{L^{2}}{2\cdot \mu \cdot V}$$

where *μ* is the carrier mobility and *L* is the device length. Ultrafast carrier mobility leads to improving $f_{\text{transit}}$; thereby, the intrinsic bandwidth of a graphene photodetector in free space was up to 262 GHz.^[^ ^290^ ^]^ As shown in **Figure** **15**a, coupling with a Si waveguide, the responsivity of graphene photodetector with asymmetric electrodes could be 100 mA W^−1^ over a broad wavelength range from 1450 to 1590 nm. Responsivity (Re) is given by^[^ ^191^ ^]^

(22) $$\text{Re}=\frac{I_{P}}{P_{\text{in}}}=\text{EQE}\cdot \frac{\lambda \left(\mu m\right)}{1.24}(A/W)$$

where $I_{P}$ is the photocurrent, $P_{\text{in}}$ is the input power, and *λ* is the wavelength of the incident light. Without external bias, a response rate exceeding 20 GHz at the telecommunication wavelength was achieved by a Si waveguide‐integrated photodetector based on graphene. The photocurrent mapping indicated the built‐in electric field between bulk graphene and metal‐doped graphene, and the photodetector worked under the PV mode (Figure 15b).^[^ ^38^ ^]^ By further minimizing the active region of graphene device, a waveguide‐integrated photodetector showed a bandwidth of 41 GHz at the wavelength of 1550 nm and could operate at a data rate of 50 Gbit s^−1^.^[^ ^291^ ^]^ Recently, the data rate of integrated graphene photodetectors could be up to 180 Gbit s^−1^,^[^ ^292^, ^293^ ^]^ exceeding the performance of the waveguide‐integrated germanium photodetector.^[^ ^294^ ^]^

Although graphene photodetectors have many advantages including fast speed and broad optical bandwidth, the gapless character limits their capability to measure small signals. Graphene photodetectors possessed larger dark currents than those photodetectors based on semiconductors, resulting in a big shot noise. Furthermore, the photocurrent of the graphene photoconductor under weak illumination would be smaller than dark current, leading to a small on–off ratio. At optical frequencies, complex surface conductivity of graphene is given by^[^ ^295^ ^]^

(23) $$\sigma (\omega )=\frac{\sigma_{0}}{2}\cdot [\tan h(\frac{\hbar \cdot \omega +2\cdot \mu_{c}}{4\cdot k_{B}\cdot T})+\tan h(\frac{\hbar \cdot \omega -2\cdot \mu_{c}}{4\cdot k_{B}\cdot T})]-i\cdot \frac{\sigma_{0}}{2\cdot \pi }\cdot \ln[\frac{{\left(\hbar \cdot \omega +2\cdot \mu_{c}\right)}^{2}}{{\left(\hbar \cdot \omega -2\cdot \mu_{c}\right)}^{2}-2\cdot {\left(k_{B}\cdot T\right)}^{2}}]+i\cdot \frac{4\cdot \sigma_{0}}{\pi }\cdot \frac{\mu_{c}}{\hbar \cdot \omega +i\cdot \hbar \Gamma }$$

where $\sigma_{0}=q^{2}/(4\cdot \hbar )$ is the universal conductivity of monolayer graphene, Γ is the phenomenological scattering rate induced by different scattering mechanisms, $k_{B}$ is Boltzmann's constant, and *T* is the temperature. As we mentioned before, $\mu_{c}$ can be tuned by carrier accumulation, and *σ* minimizes when $\mu_{c}$ locates at the charge‐neutral point (Figure 9a). To suppress dark current, one method was applied on the external electric field to tune *E*_F_ of graphene. In a graphene–Al_2_O_3_–graphene capacitor, the top graphene layer served as a gate electrode to tune *E*_F_ of the bottom graphene layer, leading to a change in resistance from about 30 to 110 Ω (Figure 15c).^[^ ^296^ ^]^ When *E*_F_ of graphene is tuned to the Dirac point, dark current minimizes. Furthermore, when *E*_F_ locates at the charge‐neutral point (the maximal resistance), the absorption coefficient maximizes because of strong interband absorption, leading to a higher responsivity. In 2016, graphene was integrated with slot waveguides whose separated silicon waveguides could serve as two bottom‐gate electrodes to dope graphene, forming a PN junction laterally, as shown in Figure 15d,^[^ ^297^ ^]^ leading to smaller dark current and other optimal performances such as a 3 dB bandwidth of 65 GHz. There are several technical difficulties to realize photodetectors based on a slot waveguide with two gate electrodes. For example, stair‐stepping‐doped Si is necessary to not only obtain Ohmic contact between electrode and Si, but also reduce optical loss induced by free‐carrier absorption, which is costly during the fabrication process. In addition, 2D h‐BN has also been demonstrated as a better dielectric than SiO_2_ for improving carrier mobility of 2DMs because of the reduced Coulomb scattering. For example, a waveguide‐integrated h‐BN/graphene/h‐BN photodetector showed both smaller dark current and a 3 dB bandwidth of 42 GHz.^[^ ^298^ ^]^

**Figure 16:**
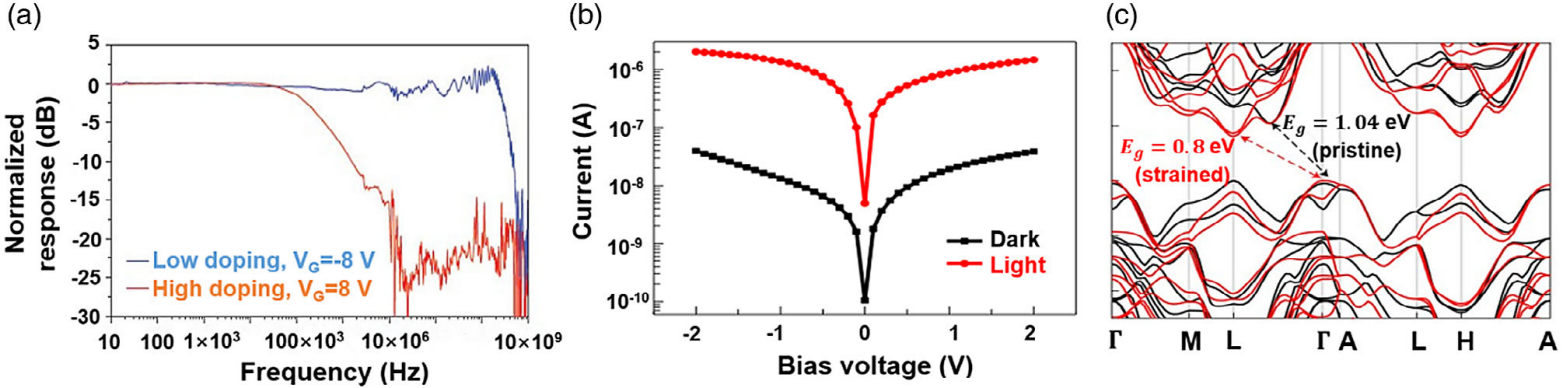
a) The photoresponse of the BP photodetector under different doping conditions. Reproduced with permission.^[^ ^299^ ^]^ Copyright 2015, Springer Nature. b) *I–V* curves of waveguide‐integrated photodetectors based on MoTe_2_. c) The band structure of pristine and strained MoTe_2_ that is calculated by first‐principles density functional theory. b,c) Reproduced with permission.^[^ ^289^ ^]^ Copyright 2020, Springer Nature.

Another direction to decrease the dark current and improve the NEP is integrating other 2DM semiconductors with a suitable bandgap. Unlike graphene, BP has a direct bandgap at any thickness and its dark current could be tuned to nanoamperes via electrical gating. The first gate‐controlled waveguide‐integrated BP photodetector was reported in 2015, and graphene was used as the transparent gate electrode.^[^ ^299^ ^]^ When BP was tuned to the charge‐neutral point, the waveguide‐integrated photodetector operated in the PV mode with high responsivity (657 mA W^−1^), low dark current (220 nA), broad bandwidth (3 GHz), and high internal quantum efficiency (50%). Otherwise, when BP was heavily doped, the photodetector operated in the BE mode and the responsivity and 3 dB bandwidth would be much lower (**Figure** **16**a), which is limited by low in‐plane lattice thermal conductivity of BP. Instability is an obvious disadvantage of the photodetector based on BP, leading to a strict limitation to device fabrication and applications. Recently, multilayer MoTe_2_ showed its advantage to realize the waveguide‐integrated photodetector at the wavelength of 1550 nm with a dark current of 13 nA (Figure 16b). The bandgap of MoTe_2_, without strain, is about 1.04 eV, and it shifts to 0.8 eV under 4% tensile strain (Figure 16c),^[^ ^289^ ^]^ which provides a good solution to realize photodetectors at telecom wavelengths. In this Article, multilayer MoTe_2_ was transferred onto a nonplanarized waveguide with a height of 220 nm to realize strain engineering. This fabrication seems not to be suitable for high‐density integrated devices and could probably result in cracks in MoTe_2_ during the transfer. More ingenious designs for strain engineering are required in the future.

**Figure 17:**
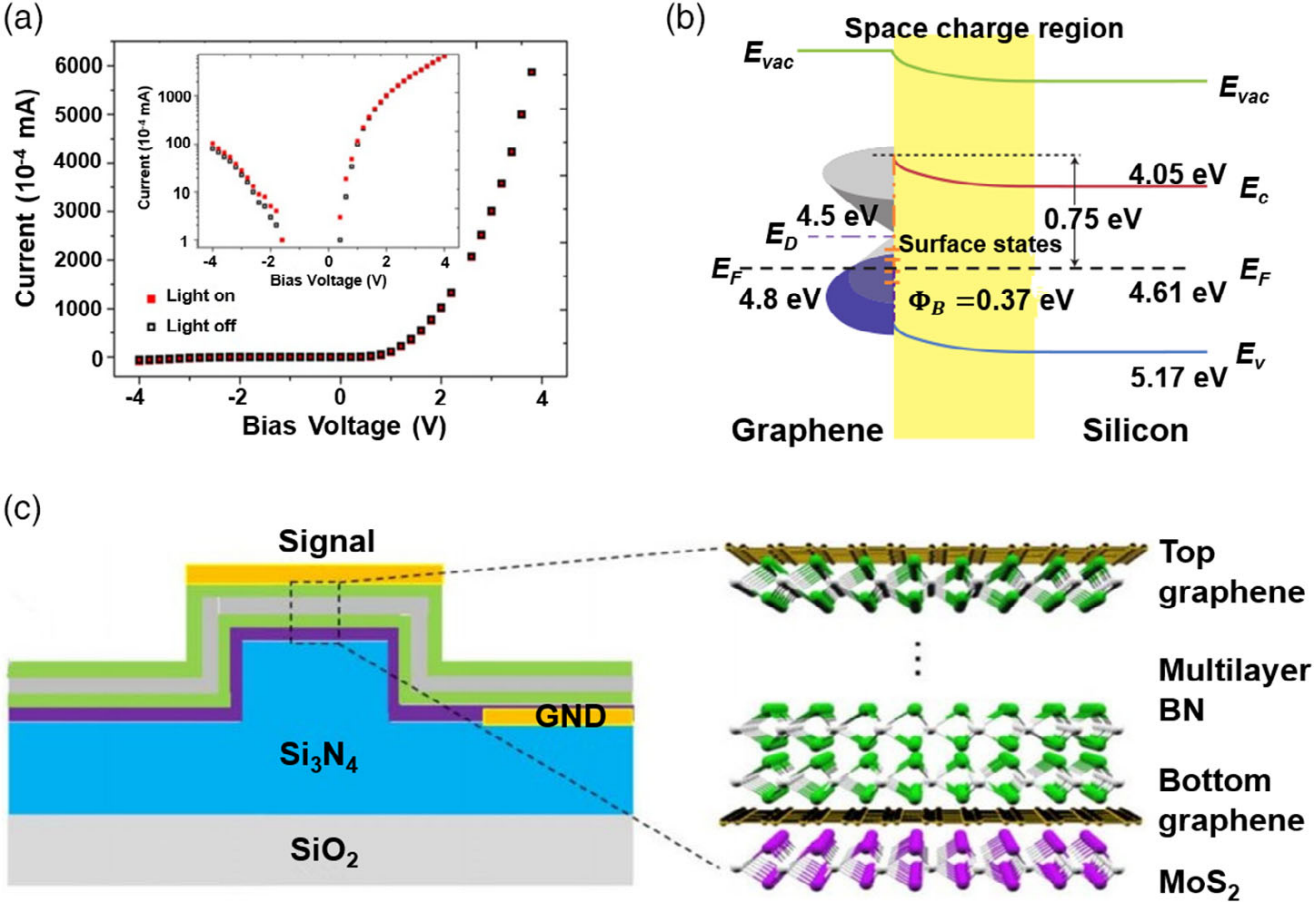
a) The *I–V* curve of a graphene/Si waveguide photodetector. Reproduced with permission.^[^ ^303^ ^]^ Copyright 2016, AIP Publishing. b) Illustration of the energy band of graphene/Si heterojunction in the dark. Reproduced with permission.^[^ ^356^ ^]^ Copyright 2013, Springer Nature. c) Schematic of a waveguide‐integrated photodetector based on van der Waals heterojunction. Reproduced with permission.^[^ ^302^ ^]^ Copyright 2019, Optical Society of America.

Van der Waals heterojunction can be used to suppress dark current and improve the device performance of 2DM photodetectors as well. In a 2DM/bulk semiconductor heterojunction device, the relationship between current and applied voltage can be expressed as^[^ ^300^ ^]^

(24) $$I=I_{s}\cdot [\exp(\frac{q\cdot \left(V-I\cdot R_{s}\right)}{\eta \cdot k_{B}\cdot T})-1]$$

where $I_{s}$ is the reverse saturation current, *η* is the ideality factor. As shown in **Figure** **17**a, the *I–V* curve of graphene/Si waveguide shows obvious rectification property. At the same reverse bias, dark current is much lower than that of the graphene photoconductor, which is attributed to the energy band (Figure 17b). At a reverse bias, the length of the space–charge region becomes longer, leading to bigger resistance. Benefitting from the fast speed of carrier drift, the response time (*t*) is up to a few nanoseconds for a graphene/Si heterojunction photodetector, which can be fitted as^[^ ^300^ ^]^

(25) $$t=2.2\cdot R_{s}\cdot (C_{0}+\frac{\epsilon_{0}\cdot \epsilon_{\text{Si}}\cdot A}{d_{0}}\cdot \sqrt{\frac{V_{\text{bi}}}{V+V_{\text{bi}}}})$$

where $\epsilon_{\text{Si}}$ is the relative dielectric constant of Si, $C_{0}$ is the structural capacitance, $V_{\text{bi}}$ is the built‐in potential of the heterojunction, and *A* is the device area. A heterojunction consisting of several different 2DMs has shown advantages for photodetectors. For example, a waveguide‐integrated photodetector based on graphene/MoTe_2_/h‐BN heterojunction was realized, where the bottom graphene was used as an electrode, and top h‐BN was used for passivation. Benefitting from the ingenious device structure, this photodetector with a low dark current exhibited a responsivity of 23 mA W^−1^ at the wavelength of 1310 nm over a large dynamic range, a bandwidth of 2 GHz, and a data rate of 1 Gbit s^−1^.^[^ ^301^ ^]^ The bandwidth more than 50 GHz has been realized with the decreasing channel length, resulting from a fast transit time,^[^ ^42^ ^]^ which provides a good solution to design high‐speed photodetectors. A waveguide‐integrated photodetector based on the MoS_2_/graphene/multilayer h‐BN/graphene tunneling heterojunction was proposed (Figure 17c). In this device, MoS_2_ worked as both the passivation layer and the doped layer to bottom n‐doped graphene. Photoinduced carriers could be separated by the built‐in electric field at the junction between n‐doped and p‐doped regions of graphene. Multilayer h‐BN, in the middle of top and bottom graphene, served as a tunneling barrier, leading to a decrease of dark current (several nA).^[^ ^302^ ^]^

Besides tuning detection materials and device structures, the optimization of photonic structures is also critical for improving the performance of 2DM photodetectors. As we discussed in the modulator section, total absorption ($A_{\text{total}}$) of photoactive materials is given by

(26) $$A_{\text{total}}=P_{\text{in}}\cdot (1-\exp(-\alpha_{p}\cdot l))$$

where *l* is the photodetector length along the waveguide, $P_{\text{in}}$ is the input power, and $\alpha_{p}$ is the absorption coefficient of photoactive materials for photodetectors. Therefore, enhancing $\alpha_{p}$ is an effective approach to improve photon‐to‐electron transition. First, microcavity resonator is a structure to capture light energy and improve optical absorption. However, an intrinsic characteristic for the resonator is its narrow optical bandwidth. Compared with the Si waveguide, photonic‐crystal waveguide serving as a medium to guide light can improve optical absorption as we mentioned earlier.^[^ ^303^ ^]^ Third, plasmonics, an effective light‐trapping structure, has been used to enhance the optical absorption of photodetectors based on 2DMs in the past few years.^[^ ^292^, ^304^, ^305^ ^]^ A photodetector based on the Schottky junction consisting of a Si waveguide and graphene/Au contact was reported.^[^ ^304^ ^]^ SPPs can be excited at the Au/Si waveguide interface,^[^ ^306^ ^]^ which showed locally improved electric fields perpendicular to the graphene and Schottky interface, leading to an improved responsivity of 370 mA W^−1^ at the wavelength of 1550 nm. Another SPP‐enhanced integrated photodetector was realized by depositing a bowtie‐shaped nanostructure metal upon waveguide and graphene. Benefitting from eight‐fold enhanced local fields, the responsivity of this device was up to 500 mA W^−1^ at the wavelength of 1550 nm and the response rate was more than 110 GHz.^[^ ^292^ ^]^ Device length reduces with the enhancement of $\alpha_{p}$, leading to lower resistance and a higher $f_{3\text{dB}}$. Although $\alpha_{p}$ can be enhanced by SPP, optical loss is inevitable because of metal absorption.

Photodetectors based on 2DMs have been systematically studied, ranging from basic physical mechanisms to methodologies in the past few years. In this section, waveguide‐integrated photodetectors based on 2DMs were discussed, and figures of merit of some typical waveguide‐integrated photodetectors are shown in **Table** **3**. In the past five years, waveguide‐integrated photodetectors based on 2DMs have made tremendous progress, but there are still many theoretical and engineering challenges to overcome. First, high‐speed light detection is a significant functionality for integrated photonic circuits. 2DMs including graphene and BP with ultrafast carrier mobility, being compatible with existing CMOS backend technology, are promising for high‐speed light detection. Limited by 2DMs and nanofabrication technologies, however, large‐scale high‐speed waveguide‐integrated photodetectors have not been demonstrated yet. Second, highly sensitive light detection with low noise is necessary for integrated photonics and many other commercial applications such as biomedical imaging. Thus integrating with light‐trapping structures and improving the ability of carrier extraction are essential and necessary. Third, flexible light detection is a significant advantage of photodetectors based on 2DMs because of its inherent mechanical flexibility for conformal integration, but integrated flexible photodetectors based on 2DMs have rarely been demonstrated. Last but not the least, narrow‐bandgap 2DMs are attractive for light detection in MIR integrated photonic applications.

**Table 3:** Figures of merit in typical waveguide‐integrated photodetectors based on 2DMs

Material and device structure	Operational wavelength [μm]	Responsivity [mA W^−1^]	3 dB Bandwidth [GHz]	Optical data links [GBit s^−1^]	Ref
Graphene on Si waveguide	1.45–1.59	108	>20	12	[38]
Graphene on Si waveguide	1.31–1.65	50	≈18	NR	[355]
Graphene/Si waveguide heterojunction	1.55–2.75	130	NR	NR	[356]
Graphene on Si waveguide	1.55	16	41	50	[291]
Graphene PN junction on Si waveguide	1.56	76	65	NR	[297]
Graphene on ChG waveguide	2.00–2.55	250	NR	NR	[43]
Graphene on Si waveguide and enhanced by SP	1.48–1.62	500	110	100	[292]
Multilayer BP on Si waveguide and tuned by a gate electrode	1.55	657	3	NR	[299]
BP on ChG waveguide	2.18	40	NR	NR	[361]
BP on Si grating and tuned by a gate electrode	3.68–4.03	2000–23000	NR	NR	[362]
Multilayer BP on Si waveguide	2	306.7	1.33	4	[363]
MoTe_2_ PN junction on Si waveguide	1.06–1.24	≈5	0.2	NR	[133]
Multilayer MoTe_2_ on Si waveguide	1.31	23	1	1	[301]

## 6 Advanced Integrated Optoelectronic Devices

2DMs have already shown advantages for designing essential devices for on‐chip integrated photonics, including light sources, optical modulators, and photodetectors. With the increasing exploration of 2DMs, recently, more fundamental physics has been investigated by exploiting 2DMs’ novel properties such as quantum character, phase transformation, magnetism, and superconductivity, promoting the development of some advanced optoelectronic devices. For example, magnetic 2DMs such as Cr_2_Ge_2_Te_6_^[^ ^307^ ^]^ and CrI_3_^[^ ^69^ ^]^ that largely decouple from substrates and allow to be tuned by electrical field^[^ ^308^ ^]^ have been discovered, which can be used to design magneto‐optic and magneto‐electric devices for integrated photonics. Phase‐change 2DMs could provide the possibility to design phase‐change switch for integrated photonics.^[^ ^309^, ^310^, ^311^, ^312^, ^313^ ^]^ Bilayer graphene with a magic angle has proved to be a superconductor and would be a good choice to design single‐photon detection. More discussions on these advanced integrated optoelectronic devices are described in detail below.

### 6.1 Quantum Emitters

Quantum emitters, playing a significant role in emerging quantum technologies such as photonic quantum computing and quantum key distribution, depended previously on wide‐bandgap semiconductors, single molecules, quantum dots, and recently on 2DMs. Among different 2DMs, quantum emitters were observed in TMDCs previously.^[^ ^314^, ^315^ ^]^ For example, monolayer WSe_2_ was found to possess excellent emitting characteristics, exhibiting a narrow line width of 130 μ eV.^[^ ^316^ ^]^ On‐chip quantum emitters based on WSe_2_ integrated with the metal waveguide were also demonstrated.^[^ ^317^, ^318^ ^]^ However, the majority of quantum emitters based on TMDCs are only working at cryogenic temperatures. Recent studies indicated that h‐BN was an alternative material for room‐temperature quantum emitters.^[^ ^319^, ^320^ ^]^ To improve the performance of quantum emitters, not only materials themselves but also the device design and fabrication technologies have to be continuously explored and optimized.

### 6.2 Phase‐Change Switch

Phase‐change switch that can dynamically tune light transmission is an essential component for integrated photonic circuits,^[^ ^321^, ^322^ ^]^ which usually relies on phase‐change materials (PCMs) such as sulfur compounds (S, Se, Te). PCMs possessed reversible structural changes that tune the electrical, optical, magnetic, and thermal properties, under external excitation. Recently, a reversible structural change has been observed in 2DMs, which can be obtained by several approaches including stress induction, electron doping besides electrostatic doping and electron transfer, and laser illumination.^[^ ^323^ ^]^ For example, the transition in monolayer MoS_2_ from metal to the insulator could be observed, which was attributed to strong electron–electron interaction achieved by high levels of doping.^[^ ^324^ ^]^ In a latter study, a transient reversible semiconducting trigonal prismatic (2H) to metallic octahedral (1T) transition occurred because of the plasmonic hot‐electron transfer.^[^ ^325^ ^]^ Besides MoS_2_, other TMDCs such as MoTe_2_ and CoSe_2_ have shown phase change already. For instance, monolayer MoTe_2_ has exhibited a reversible structural phase transition by electrostatic doping, which was the first experimental result by this approach.^[^ ^326^ ^]^ Recently, electronic devices such as nonvolatile phase‐change transistor,^[^ ^327^ ^]^ resistive memories,^[^ ^328^ ^]^ and devices for neuromorphic computing^[^ ^329^ ^]^ have been demonstrated. Exploring these electronic devices based on 2D PCMs would promote the development of optical phase‐change switching.

### 6.3 Magneto‐Optic Isolator

Magneto‐optic isolators that can block back‐reflected light transporting and thus decrease the bit error rate in integrated photonic circuits are significant components for integrated photonics.^[^ ^330^, ^331^, ^332^ ^]^ An ideal optical isolator is supposed to possess several parameters including monolithic integration, low insertion loss, large isolation bandwidth, and high isolation ratio, which have been achieved by several approaches such as nonlinear effect^[^ ^333^ ^]^ and active refractive index modulation.^[^ ^334^ ^]^ Among these different optical isolation approaches, the magneto‐optic effect seems to be the most attractive way. With the effort of the integrated photonics community, considerable progress on the magneto‐optic isolator has been made.^[^ ^335^, ^336^, ^337^ ^]^ For instance, an on‐chip magneto‐optic isolator was reported recently, which operated both the transverse electric (TE) and transverse magnetic (TM) mode with high performance.^[^ ^338^ ^]^ As the aforementioned description, 2DMs possess distinct advantages in monolithic integration, which probably provide new developments for a magneto‐optic isolator in the future. Except for these devices discussed earlier, 2DMs also offer the possibility to realize other single photodetection devices by 2D superconductors.^[^ ^131^, ^339^ ^]^

## 7 Conclusion and Outlook

**Figure 18:**
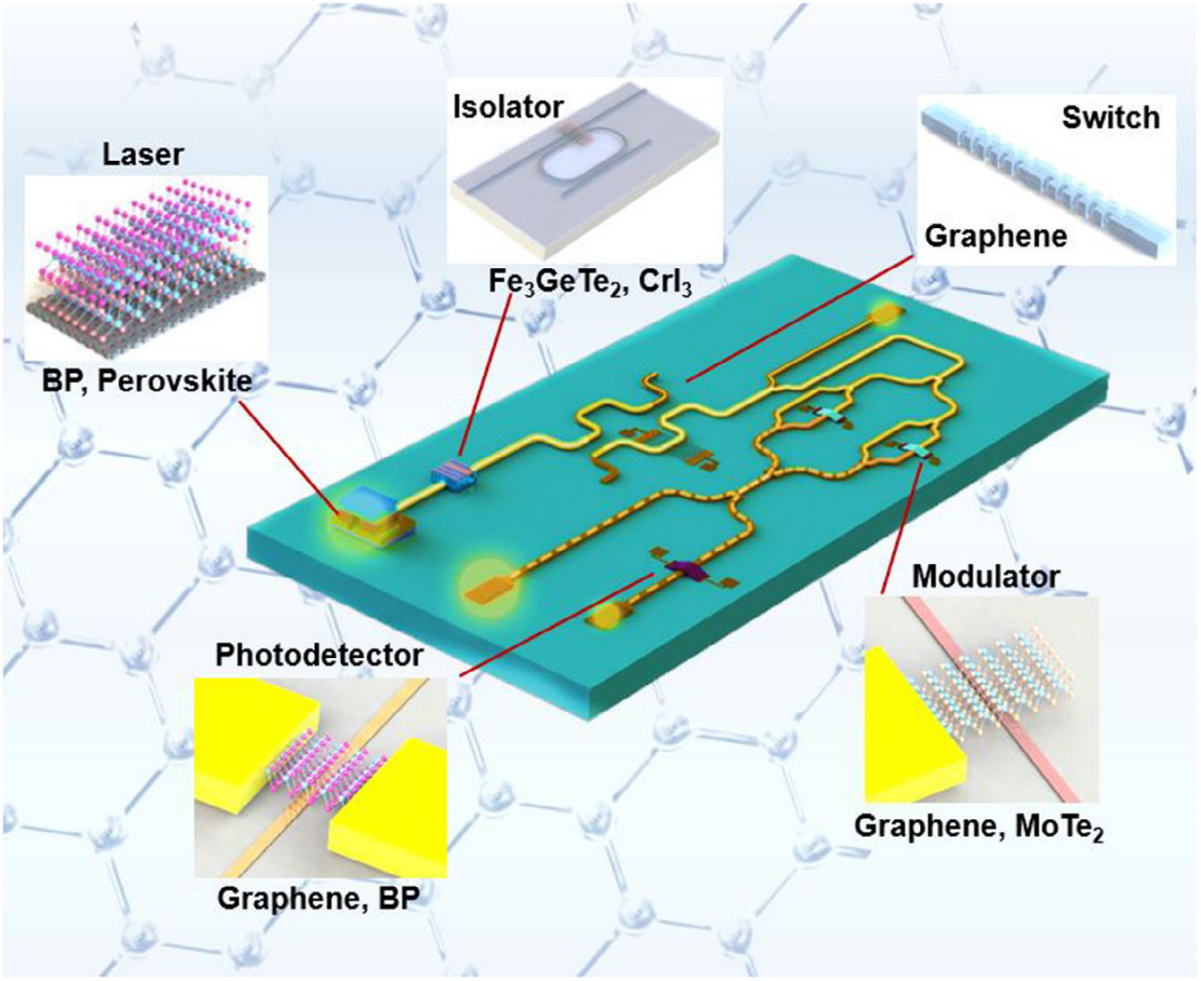
A basic schematic of an integrated photonic circuit with active optoelectronic devices including a light source, isolator, switch, modulator, photodetector.

Plenty of 2DMs have been widely investigated since the discovery of graphene. Based on these 2DMs, many proof‐of‐concept optoelectronic devices have already been demonstrated after more than a decade from the efforts all over the world. Up to now, 2DMs‐based light sources with laser operating wavelengths spanning from the visible to MIR regime,^[^ ^80^, ^158^, ^174^, ^340^ ^]^ the graphene modulator with a switching time of 260 fs,^[^ ^40^ ^]^ and the photodetector with a response time faster than 50 fs^[^ ^341^ ^]^ have been gradually demonstrated, as well as some novel advanced integrated devices including, but not limited to, isolators; phase‐change switches may be realized in the near future, making it possible to construct a complete photonic circuit (**Figure** **18**) through a combination of the state‐of‐art devices with different functions.

However, there remain difficulties in practical implementation limited by the currently unresolved theoretical and engineering problems associated with 2DM integrated devices. The first one is to synthesize a variety of large‐area, high‐quality, and single‐crystalline 2DMs for different functional devices. Recent studies on graphene and h‐BN show significant progress,^[^ ^342^, ^343^ ^]^ but the synthesis of large‐area, high‐quality uniform 2DMs with superior and unique optical, electrical, and magnetic properties is, however, still lacking. Meanwhile, it is profoundly meaningful to devise material synthesis strategies, so large‐area lateral and vertical heterojunctions in 2DMs can be achieved in situ directly, considering the importance of heterojunctions in both fundamental physics and device applications. The second challenge involves transferring 2DMs onto target substrates without producing any cracks, wrinkles, or contamination, which is crucial for device performance as the properties of the 2DMs are very sensitive to surface defects and contamination. Therefore, on the one hand, more optimal transfer approaches are supposed to be developed.^[^ ^344^, ^345^ ^]^ On the other hand, synthesizing 2DMs directly onto the target substrate may provide an alternative way^[^ ^346^ ^]^ to avoid the transfer step. Despite that, only a few 2DMs have been applied to waveguide‐integrated photonic devices exhibiting excellent performance. The current challenge lies in how to maintain the 2DM properties during the whole fabrication process; otherwise, the ultimate device performance could be severely degraded. As previously discussed, most of the devices rely on 2DM transfer on prepatterned substrates, and the abrupt change in step height could probably damage the integrity of 2DMs. In addition, it is hard to exempt from surface contamination and damage induced by nanofabrication techniques involving plasma etching, sputter, and ion beam‐assisted deposition and high‐temperature annealing in the process, which would lead to a decrease in electrical and optical properties of 2DMs. Ongoing improvements include monolithic integration of devices on 2DMs and the insertion of dielectric polymer, glasses, and self‐assembled monolayer molecular regions at the 2DM/substrate interface to ensure 2DMs with preserved carrier mobility and lifetime through minimizing the Coulomb scattering^[^ ^347^ ^]^ from surface defects and contamination. Apparently, besides nondestructive fabrication approaches, new and innovative photonic designs are also necessary and critical to expand the application of 2DM integrated devices. Especially for some emerging materials possessing unique optical, electrical, and magnetic properties, various device functions can be achieved utilizing creative and rational structure designs and be thus applied in emerging fields such as flexible and MIR integrated photonics.

Overall, benefiting from their exceptional material properties, 2DMs have attracted increasing attention in both traditional and emerging applications of integrated photonics.

## Conflict of Interest

The authors declare no conflict of interest.
